# Infinity additive manufacturing of continuous microstructured fiber links for THz communications

**DOI:** 10.1038/s41598-022-08334-6

**Published:** 2022-03-16

**Authors:** Guofu Xu, Kathirvel Nallappan, Yang Cao, Maksim Skorobogatiy

**Affiliations:** Department of Engineering Physics, École Polytechnique de Montréal, Montréal, H3T 1J4 Canada

**Keywords:** Terahertz optics, Fibre optics and optical communications, Polymers

## Abstract

In this work, a novel infinity 3D printing technique is explored to fabricate continuous few-meter-long low-loss near-zero dispersion suspended-core polypropylene fibers for application in terahertz (THz) communications. Particular attention is paid to process parameter optimization for 3D printing with low-loss polypropylene plastic. Three microstructured THz fibers were 3D printed using the standard and infinity 3D printers, and an in-depth theoretical and experimental comparison between the fibers was carried out. Transmission losses (by power) of 4.79 dB/m, 17.34 dB/m, and 11.13 dB/m are experimentally demonstrated for the three fibers operating at 128 GHz. Signal transmission with bit error rate (BER) far below the forward error correction limit (10^–3^) for the corresponding three fiber types of lengths of 2 m, 0.75 m, and 1.6 m are observed, and an error-free transmission is realized at the bit rates up to 5.2 Gbps. THz imaging of the fiber near-field is used to visualize modal distributions and study optimal fiber excitation conditions. The ability to shield the fundamental mode from the environment, mechanical robustness, and ease of handling of thus developed effectively single-mode high optical performance fibers make them excellent candidates for upcoming fiber-assisted THz communications. Additionally, novel fused deposition modeling (FDM)-based infinity printing technique allows continuous fabrication of unlimited in length fibers of complex transverse geometries using advanced thermoplastic composites, which, in our opinion, is poised to become a key fabrication technique for advanced terahertz fiber manufacturing.

## Introduction

The terahertz (THz) spectral range (0.1–10 THz) has recently attracted much attention because of many potential applications in sensing^1,2^, imaging^3,4^, security^5,6^, and communications^7,8^. Particularly, the THz communications are expected to provide the solution for exponentially increasing IP data traffic in the near future with bitrates of at least several tens of gigabytes per second per user. In this scenario, the wireless links for transmitting large data volumes are a particularly convenient modality for mobile end-users. According to the Shannon theorem, for a given signal-to-noise ratio, the maximum capacity of the channel is limited by its bandwidth. To support higher bit rates, shifting the carrier frequency towards a higher frequency range (THz spectral band and hence higher bandwidth) is one promising solution. Nevertheless, there are several challenges in realizing the free space wireless THz links. Firstly, atmospheric absorption of the THz waves is strongly sensitive to the environmental conditions, which could potentially make such links unreliable for all-weather operation^9,10^ (see Supplementary Materials Section S1, Note 1). Additionally, due to the higher frequency of THz waves compared to microwave radiation, they are more prone to scattering on various obstacles and structural imperfections, thus making non-line of sight THz communication challenging^11^. In turn, the signal interference effects can become pronounced in the ultra-dense THz wireless networks^12^. Moreover, due to self-diffraction of the electromagnetic waves, the free space path loss increases as a square of the distance between the transmitting and receiving antennas, even in absence of the atmospheric absorption, which can be of importance for longer THz links or smaller antenna sizes (see Supplementary Materials Section S1, Note 2). Finally, strong directionality of the THz beams demands tight alignment tolerances between the transmitting and receiving antennas for high-quality THz wireless links^13^ (see Supplementary Materials Section S1, Note 3). In this respect, the THz waveguides/fibers can be an alternative solution to the medium-length (~ 10 s of meters) THz communications links due to the fiber's small footprint and flexibility, as well as ease of handling and installations even in complex geometrical environments^14,15^. Moreover, many properly encapsulated THz fibers are largely immune to various environmental variations. This is because, by design, the modal fields in such fibers are localized well inside of the fiber outer shell. This makes such fibers easy to handle, install and service as they tolerate touching without the danger of core contamination, as well as installation using mounts in direct contact with the fiber surface. When properly sealed, such fibers also resist variations in the transmission properties under the influence of atmospheric factors such as changes in air quality, humidity, and other factors. Moreover, plastic THz fibers are also immune to external electromagnetic interference and eavesdropping promising reliable and secure communications^16^. Ultimately, seamless integration of THz wireless links with THz fiber-assisted links can offer reliable performance in the next generation of communication systems. In such systems, one can envision, for example, that a THz fiber network will be installed within the geometrically complex communication environment (such as a multistory building) to provide reliable points of THz wireless access within smaller and less complex communication environments (such as individual offices).

To realize efficient THz fiber-assisted communication links, the THz fibers must feature low transmission loss, low bending loss, low group velocity dispersion (GVD), high coupling efficiency, as well as good mechanical stability, and low sensitivity to the various environmental variations^14^. The low-loss guidance in THz fibers is generally achieved by adapting designs that allow a significant fraction of THz light to propagate in the low-loss gaseous cladding that is encapsulated inside of a certain mechanical superstructure. Moreover, versatility, complexity, and cost of the fiber fabrication techniques are important factors for their ultimate acceptance by the industry and practical deployment. While the gold standard for fabrication of the kilometer-long optical fibers is a drawing technique, THz range offers additional opportunities for fiber manufacturing as one needs only several tens of meter-long THz fibers. In this paper, we argue that 3D printing, and its variant—infinite 3D printing, offer a promising fabrication method for continuous multi-meter long fibers with complex cross-sections. In fact, one of the key limitations of standard 3D printers for THz fiber development is a limited build volume. Recently, a novel additive manufacturing approach known as infinite-Z or limitless belt or simply infinite 3D printing was developed that enables fabrication of 3D structures without any length limitations along a single direction^17–19^. This method opens the possibility of fabricating THz fibers or even complete fiber devices of unlimited length and arbitrary complex 2D and 3D profiles.

A standard way of Microstructured Polymer Optical Fibers (MPOF) fabrication is a fiber drawing technique. During drawing, a tip of the structured fiber preform is softened and then pulled into a fiber featuring a constant transverse profile along its length. With the recent advances in Rapid Prototyping techniques, such as stereolithography (SLA) and fused deposition modeling (FDM), the direct printing of complex fiber preforms and fiber has been gaining popularity as such robust and relatively inexpensive techniques eliminate the need for expensive fiber drawing infrastructure. In fact, fiber drawing is highly efficient in the fabrication of kilometer-long fibers with outstanding wall roughness control, which is only limited by thermal fluctuations on a softened plastic or glass surface^20–22^. A typical drawing speed for fiber fabrication ranges from several cm/s to tens of m/s, with wall roughness that can be smaller than a nanometer. It is indeed a method of choice for fibers designed for operation at shorter wavelengths from visible to mid-IR. Although the infinity 3D printing method also allows for unlimited-length fiber fabrication, it is much slower than fiber drawing with typical fiber fabrication speeds of ~ 0.1 mm/s. At the same time, the useful length of THz fibers is limited by high losses of the fiber materials and is typically shorter than ~ 10 m, which brings such fibers within the realm of 3D printing. Moreover, in certain aspects, the infinity 3D printing technique is superior to fiber drawing. Particularly, at longer wavelengths (THz, microwaves), fiber diameters tend to be large (on the order of cm). Continuous fabrication of cm-diameter fibers is, in fact, problematic for a fiber drawing technique, and in this limit, one would rather resort to extrusion. The problem stems from the fact that diameters of furnaces used in plastic and specialty glass fiber fabrication are usually in the 5–10 cm range. When drawing large diameter fibers, strong radial temperature gradients within the fiber make the process challenging to control. Extrusion has problems of its own due to the complexity of dye design for microstructured fiber fabrication^23^. In contrast, it is standard for 3D printing techniques (such as SLA and FDM) to fabricate 3D-patterned cm-size structures, thus making it a robust method for the fabrication of THz fibers and even complete fiber devices with extremely complex transverse geometries, which is difficult, if not impossible, for fiber drawing. We note also that although extremely complex fibers (photonic crystal and photonic bandgap fibers, etc.) have been drawn and widely reported, process development for the fabrication of such fibers is long and labor-intensive, while the infrastructure investment is considerably more significant (~ 1 M$) than for 3D printing (~ 10 K$). We, therefore, believe that the ability of infinity 3D printers to produce multi-meter long large-diameter fibers of virtually unlimited 3D complexity is a great motivation for further exploration of this approach for the fabrication of fibers and fiber devices operating at longer wavelengths (far-IR, THz, microwave).

As a reminder, the SLA technique uses layer-by-layer selective photopolymerization, offers high resolution (~ 50 µm), and is capable of making structures of virtually unlimited 3D complexity. Due to the high resolution of the SLA technique, fabricated THz structures can operate even at higher frequencies ~ 0.5–1 THz as the corresponding wavelengths (600–300 µm) are still much longer than the SLA resolution. At the same time, photopolymer resins used in this process are relatively lossy (> 4000 dB/m) in the THz range, consumables are expensive, while build volumes are limited to ~ 10 cm in every direction, thus allowing fabrication of only short waveguide sections. Due to its high spatial resolution, the SLA technique was successful in demonstration of many advanced waveguides such as Kagome photonic crystal hollow-core waveguides^24^, pentagram hollow-core anti-resonant waveguides^25^, double pentagonal nested hollow-core waveguides^26^, hollow photonic bandgap waveguides with hyperuniform disordered reflectors^27^, two-wire plasmonic THz circuits^28^, hollow-core Bragg waveguides with integrated fluidic channels^29^, and many others.

An alternative to the SLA technique is an FDM technique that lays out layer-by-layer of a thermopolymer melt squeezed out from a hollow nozzle. Due to the thermo-mechanical nature of the process, FDM resolution is limited by the nozzle opening that normally exceeds 200 µm. At the same time, FDM techniques can use a variety of polymer materials in the THz range (0.1–0.3 THz) featuring medium-losses (~ 40–2000 dB/m) Poly(methyl methacrylate) (PMMA)^30^, Polyethylene Terephthalate Glycol (PETG)^31^, Acrylonitrile Butadiene Styrene (ABS)^32,33^, Polycarbonate (PC)^34^, Polylactic Acid (PLA)^1^, or low-losses (~ 4–200 dB/m) such as Polystyrene (PS)^35^, High Density Polyethylene (HDPE)^36^, Cyclic Olefin Copolymer (also known as TOPAS)^37^ and Polypropylene (PP)^14,38^. Moreover, build volumes of FDM systems can be as big as 1 m in every direction. Therefore, FDM technique is considered promising for the fabrication of THz waveguides and components for operation at lower THz frequencies ~ 0.1–0.3 THz, where operation wavelengths (~ 3–1 mm) are still much longer than the FDM resolution (~ 200 µm). Many MPOFs have been recently demonstrated using FDM technique^1,33,34,39,40^, the experimental transmission losses of these MPOFs were generally found to be on the order of hundreds of dB/m (by power) in the range of 0.1–0.5 THz, with the corresponding fiber diameters and lengths in the range of ~ 1–4 cm and ~ 8–15 cm respectively (see Table 1). In addition, there are several recent works that demonstrate the drawing of polymer fibers using the 3D-printed microstructured preforms using FDM technique^30–32,41,42^. However, due to low preform quality, strong fiber and preform deformations were reported with only short fiber lengths (usually tens of cm) of consistent geometry drawn.

**Table 1:** Comparison of FDM-based 3D printed THz MPOFS.

Ref	Diameter (mm)	Length (cm)	Loss (dB/m)	Frequency (THz)
^1^	~ 36.5	~ 5–12.5	~ 100–500	0.18–0.32
^33^	~ 25	~ 10	~ 30–150	0.1–0.4
^34^	~ 24	~ 8.7	~ 102–104	0.15–0.5
^39^	~ 23	~ 9.3	~ 60–160	0.1–0.3
^40^	~ 45	~ 90	~ 13	0.1
This work	~ 8	~ 140–250	~ 3–24	0.11–0.15

Compared to these works, fibers described in this work feature small diameters, long lengths, and much lower transmission losses. More importantly, we show that such fibers can be fabricated using a continuous printing process, which allows forgoing the use of multiple mechanical splicers to obtain longer fiber strands. Superior optical properties of our fibers are due to the use of polypropylene (PP) polymer that features an almost constant refractive index and one of the lowest absorption losses in the THz regime. However, there are several challenges such as heavy warping, difficulty to adhere to the printing bed, etc., which makes it difficult to use this material as the filament for 3D printing. Therefore, an in-depth printing process optimization is a key step when using PP as a fiber material. Due to these reasons, only a few microstructured THz waveguides using PP have been reported in the literature to date. Among those, the solid-core THz waveguides are the easiest to fabricate, while their transmission losses are typically high and close to those of the fiber materials. However, by using low-loss polymers (ex. PP) and subwavelength size core, the modal transmission losses can be greatly reduced by pushing the modal field out of the lossy core and into the low-loss air cladding^14,43–46^. This enables the fabrication of high-performance THz waveguides and components for THz communications. Furthermore, subwavelength solid core can be encapsulated inside of the hollow support structure to reduce the effect of the environment and to improve the convenience of handling^47^.

In this work, we perform for the first time a comparative analysis of standard and infinite FDM printing to manufacture long and geometrically complex fibers for THz communication applications. Particularly, we fabricate a microstructured fiber that features a subwavelength core suspended in the air by three thin bridges in the middle of an encapsulation tube that allows convenient handling of such fibers without perturbing the modal fields. Moreover, the fiber is designed to operate at the point of zero dispersion at the carrier frequency of 128 GHz and is made of a low-loss Polypropylene polymer. This fiber structure is chosen as a benchmark for the comparative analysis of FDM techniques as it features complex transverse geometry (thin bridges, large encapsulation tube), complex guidance mechanism (total internal reflection in the core and radiation leakage through the bridges), complex dispersion-managed design, and challenging material (PP) for FDM technique. In the end, we find that, indeed, the newly developed infinity FDM technique is well capable of fabricating such complex fibers, while significant investment into manufacturing process improvement and optimization is still necessary to make their performance compatible with standard FDM printed fibers.

## Results

This section is organized as follows: Firstly, we introduce the design of suspended-core microstructured fiber followed by the parameter optimization for 3D printing with PP polymer. Secondly, the fabrication process of the proposed fiber using standard and infinity 3D printers is detailed. Thirdly, we present the theoretical study of the fiber modal structure and their excitation efficiencies, modal optical properties including straight and bent fiber losses, group velocity dispersion, as well as projected information capacity of the fiber links. Finally, the experimental characterization of the fiber optical properties, modal imaging, as well as Bit Error Rate measurements of various fiber links as a function of the data bitrate are detailed.

### Fiber design and fabrication with standard and infinity 3D printers

**Figure 1:**
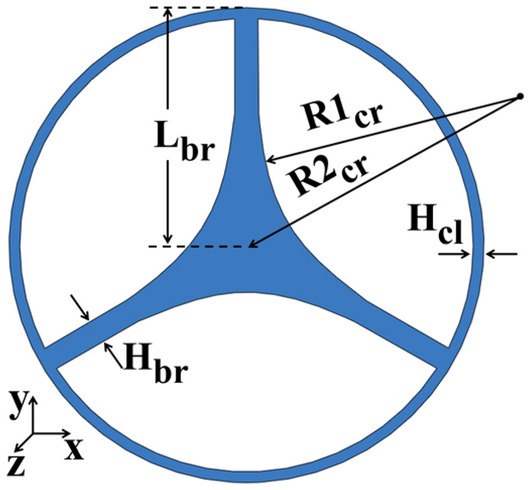
Schematic of the cross-section of a suspended core fiber.

The schematic of the proposed fiber is shown in Fig. 1. The blue regions represent the PP material, while the white regions are air. The fiber design was optimized to enable low-loss, near-zero dispersion operation at the ${\nu }_{c}=$ 128 GHz carrier frequency, which is the frequency of optimal performance of our THz communications setup detailed in our prior study^14^ (see Supplementary Materials Section S2 for details on fiber optimization). Thus optimized fiber features a negative curvature solid core of inscribed circle diameter of ~ 1.61 mm suspended by three supporting bridges of ${H}_{br}$ = 0.4 mm width. The cladding region is formed by three air holes with radii ${R1}_{cr}$ = ~3.7 mm, which are symmetrically distributed around the fiber center. The distance between the fiber center and the air hole center is ${R2}_{cr}$ = 4.5 mm. The outer fiber diameter is 8.0 mm and the cladding thickness is ${H}_{cl}=$ 0.2 mm.

In what follows, we use the Fused Deposition Modeling (FDM) to fabricate THz fibers using Polypropylene plastic that features one of the lowest losses in the THz spectral range and is translucent in the visible. The first task when 3D printing THz fibers is to optimize the printing process by minimizing the amount of trapped air in the fiber bulk regions and to reduce surface roughness. After a comprehensive printing process optimization (see Supplementary Materials Section S3 for details of printing process optimization), the following optimal PP printing parameters were found: infill flow rate of 110%, infill speed of 30 mm/s, layer height of 0.15 mm, the first layer printing speed of 50 mm/s, the inner shell printing speed of 70 mm/s, overlap of 5%, extrusion temperature of 240 °C and the built plate temperature of 95 °C.

**Figure 2:**
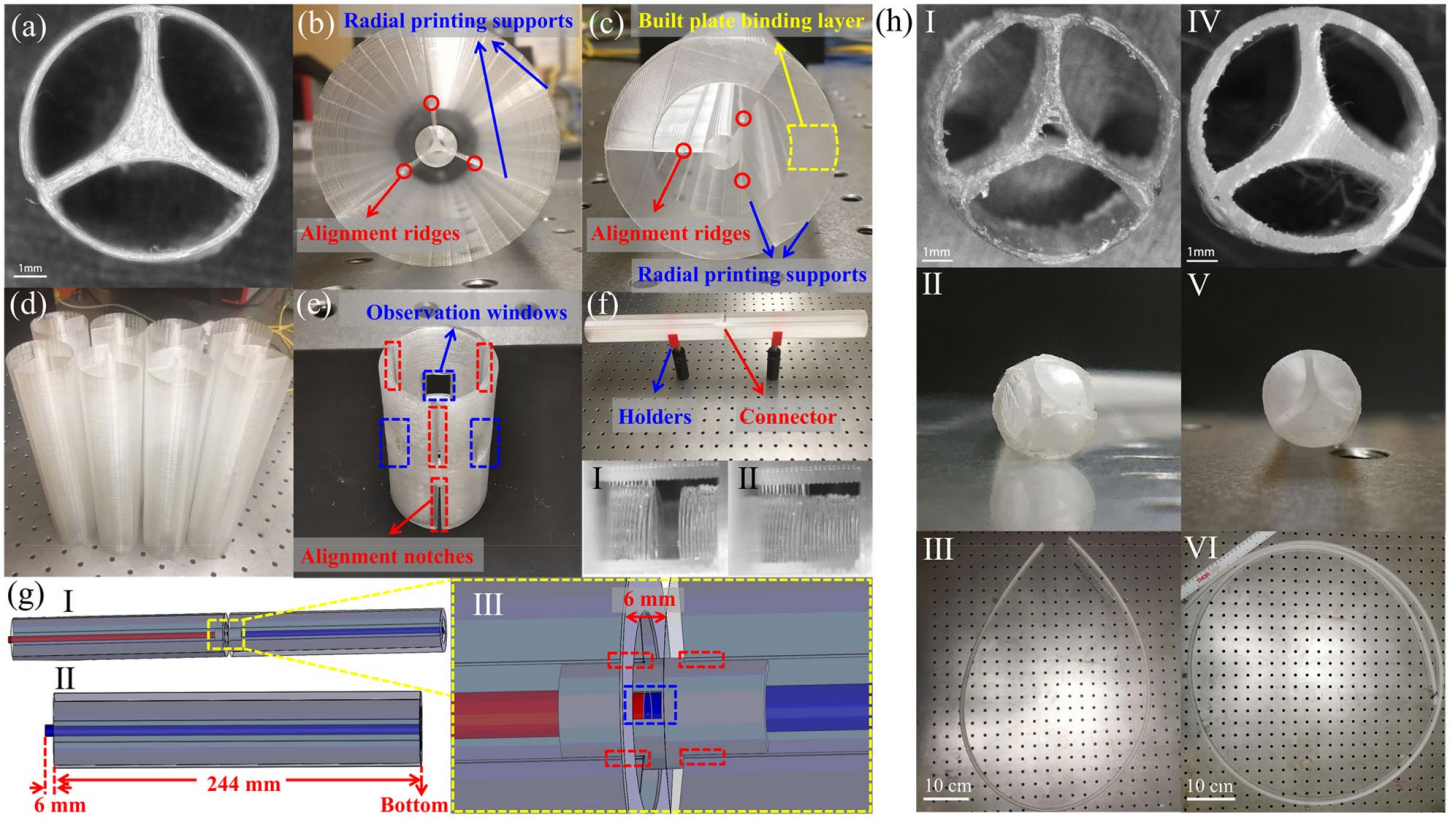
(**a**) Cross-section of the FDM printed fiber with printing supports removed. (**b**) As printed fiber section (top view) with radial printing supports and inter-section alignment ridges. (**c**) As printed fiber section (bottom view) with radial printing supports, a built plate binding layer, and alignment ridges. (**d**) Eight of the 25 cm-long fiber sections are to be combined into a 2 m-long fiber. (**e**) The connector and its 3D schematics. (**f**) Two fiber sections are connected using one connector and two holders. Insets: I (disconnected) and II (connected)—microscope view of the connection between two fiber sections. (**g**) 3D Schematics of (I) the interconnection between two fiber sections and (II) one single fiber section. (III) An enlarged schematic of the connector region featuring alignment elements (red dotted regions) and observation windows (blue dotted region). (**h**) Two several meter-long fibers were printed using an infinity FDM printer. (I) (IV) Microscopy images of the fiber cross-sections, (II)(V) side views, and (III)(VI) top views of the continuous 1.4 m-long (defect core) and 2.5 m-long (solid core) fiber sections.

Figure 2a presents the microscope image of the fiber cross-section printed using the FDM technique and design parameters mentioned earlier. We refer to such fibers as “standard” in the rest of the paper. The 25 cm-long fiber section is printed with a printer in a vertical direction aligned with the fiber length. To mechanically stabilize printing tall slender sections, a thin (0.2 mm) outer shell and three thin bridges (0.4 mm) are added on the outside of the fiber. The outer shell bridges are made 6 mm shorter than the fiber core (25 cm) in order for the cores of the two adjacent fibers to touch inside of the fiber connector shown in Fig. 2b, g. While the mechanical support structure can be easily cut from the fiber, we leave it in place to provide alignment and support during experimental measurements. An annular built plate binding layer at the bottom of the model is used to further stabilize printing (Fig. 2c), while PP tape is applied to the built plate to promote better adhesion. Finally, thus printed 8 sections (Fig. 2d) are assembled into a 2 m-long waveguide using mechanical splicing with the help of separately printed connectors (Fig. 2e) that fit on the outside of the fiber cladding. To this end, each side of the fiber sections features three alignment ridges (Fig. 2b, c) that fit the appropriate notches in the connector, resulting in seamless transitions from one fiber section into the other with cores, bridges, and cladding aligned up to ~ 50 microns positional precision with respect to each other. In Fig. 2g, f, we show schematics of the two joined waveguide sections and a connecting region.

When using standard FDM printers, fiber length is limited by the printer's linear dimensions (usually smaller than 0.5 m), while longer fiber sections must be assembled from shorter individually printed sections using connectors. Recently, a new class of FDM printers known as “infinity printers” has been developed. Such printers are designed to produce longer parts or provide continuous manufacturing of smaller parts. They use continuous printing on a slowly moving belt (which serves as a build plate) using a 45° inclined extruder. In collaboration with BlackBelt 3D BV Inc., two PP fibers (1.4 m and 2.5 m) were fabricated using their flagship infinity printer with the fiber geometry presented earlier Fig. 2h I and IV], and materials processing parameters that are similar to those used in a standard FDM printer. Images of the resultant fiber cross-sections, as well as fiber side and top views, are shown in Fig. 2h. The first 1.4 m-long fiber was printed directly on the belt without using any support and resulted in a hole defect that is persistent and uniform along the whole fiber length (1.4 m); we refer to such fiber as a “defect core” in the rest of the paper. The second 2.5 m-long fiber was printed by adding a thin rectangular binding layer between a fiber and a belt, thus improving the positional stabilization of the printing process; we refer to this fiber as “solid core” in the rest of the paper. For the two defect/solid core fibers their outer diameters are ~ 8.2/ ~ 8.0 mm, their inner cladding diameters are ~ 7.7/ ~ 7.0 mm, the smallest thicknesses of the three bridges for the two fibers are ~ 0.5/ ~ 0.6 mm, while their corresponding core sizes are ~ 1.5/ ~ 1.9 mm*.* Overall, we note that the geometrical structure of all the fibers(including the defected core one) is consistent along the entire fiber length and is reproducible as all the fabrication parameters are automatically controlled by the 3D printer via PID loops. As an example, all the processing temperatures (down to the atmospheric temperature inside the printer enclosure) are constant to ~ 1 °C in the professional FDM printers. The uniformity of all the three fibers reported in this work was verified during the cut-back measurements by dissecting ~ 2 m samples every ~ 10–25 cm while observing almost identical cross-sections. Similarly, restarting the printing process results in virtually identical fibers as long as the same plastic filament is used. When changing the filament batch, especially when migrating to a different filament supplier, one generally needs to reoptimize somewhat the printing process.

The actual fiber fabrication speed is greatly influenced by the fiber diameter, the complexity of its geometry, and the various setting of a 3D printer. Thus, for the standard 3D printer, it took ~ 60 h to print eight 25 cm-long (2 m in total) fiber sections, which correspond to an effective printing speed of ~ 0.01 mm/s. For the infinite printer, it took ~ 10 h to print ~ 1 m-long fiber sections. It is noted that a large supporting structure was required for the stable fabrication of the fiber sections along the vertical direction. On the other hand, infinite 3D printing produces fiber in the horizontal direction, thus requiring smaller or none of the supporting structures to provide stable printing. As a result, the production efficiency of similar fibers can be increased 3–6 fold with the effective printing speeds potentially approaching ~ 0.1 mm/s. Note that the hole defect in the 1.4 m-long fiber is persistent and uniform along the fiber length as evidenced from the fiber cross-sections taken along the whole fiber length (see Supplementary Materials Section S3 for more details), and is not a “failed attempt” at fiber printing. The defected core fiber is indeed a uniform fiber, similar to the two others presented in this paper. The defect appears due to a particular choice of the printing speed and filament temperature, and effectively results in the new type of fiber. The inclusion of the 1.4 m-long fiber into this paper aims at making a point that the choice of printing parameters can significantly affect the printed structure. So, for example, if a uniform hole in the fiber core is needed, it can be fabricated either via its integration into a 3D CAD model or via the choice of printing parameters.

In what follows, we aim at showing that both standard FDM-printed and properly connectorized fiber sections, as well as single strands of infinity FDM-printed fibers, can be used to realize multi-meter THz fiber-assisted communication links. Both of these methodologies have their own advantages and disadvantages. At this point, it seems that while using infinity printing, the main advantage is in its ability of a single-step fabrication of long continuous fiber links, at the same time the quality of its prints seems to be inferior to those of standard FDM printers with further thorough optimization of the infinity printing process for THz fiber manufacturing still in order (see Supplementary Materials Section S3 for comparison of surface roughness between fibers).

### Theoretical analysis of fibers fabricated using standard and infinity 3D printing

The following acronyms are used in the rest of the paper to simplify notation. Thus, fibers manufactured using infinity FDM printing are noted as either infinity defect core fiber (InfDefCor) or infinity solid core fiber (InfSolCor), while the ones fabricated using standard FDM printing are called standard solid-core fibers (StdSolCor). The dimensions of the three fabricated fibers are summarized in Table 2.

**Table 2:** Fiber dimensions.

Fiber	Outer diameter (mm)	Inner diameter (mm)	Bridge width (mm)	Core size
StdSolCor	8.0	7.6	0.4	1.6
InfDefCor	8.2	7.7	0.5	1.5
InfSolCor	8.0	7.7	0.6	1.9

#### Study 1: modal structure of the straight fibers

The three experimentally realized fibers were numerically studied using finite element COMSOL Multiphysics software. The ideal cross-section shown in Fig. 1 was used to model the optical properties of the StdSolCor. For the InfDefCor and InfSolCor, the two-dimensional models of the fiber cross-section were built using high-resolution microscope images of the corresponding fiber cross-sections imported into COMSOL. For all the fibers, we assume that the cladding is air. Effective refractive index ${n}_{PP}$ and absorption coefficient by power ${\alpha }_{PP}$ of the Polypropylene plastic were taken from our prior study^14^, and in the frequency range of $\nu$ (0.1–0.15 THz) they can be fitted as ${n}_{PP}$ = 1.485 and ${\alpha }_{PP} [dB/m]=236.31 {\nu }^{2}-37.75 \nu +3.32$, where frequency $\nu$ is in [*THz*]. In our simulations, we focus on the fiber key optical parameters such as modal loss, excitation efficiency, bending loss, and group velocity dispersion that directly impact link transmission length and link bitrate.

**Figure 3:**
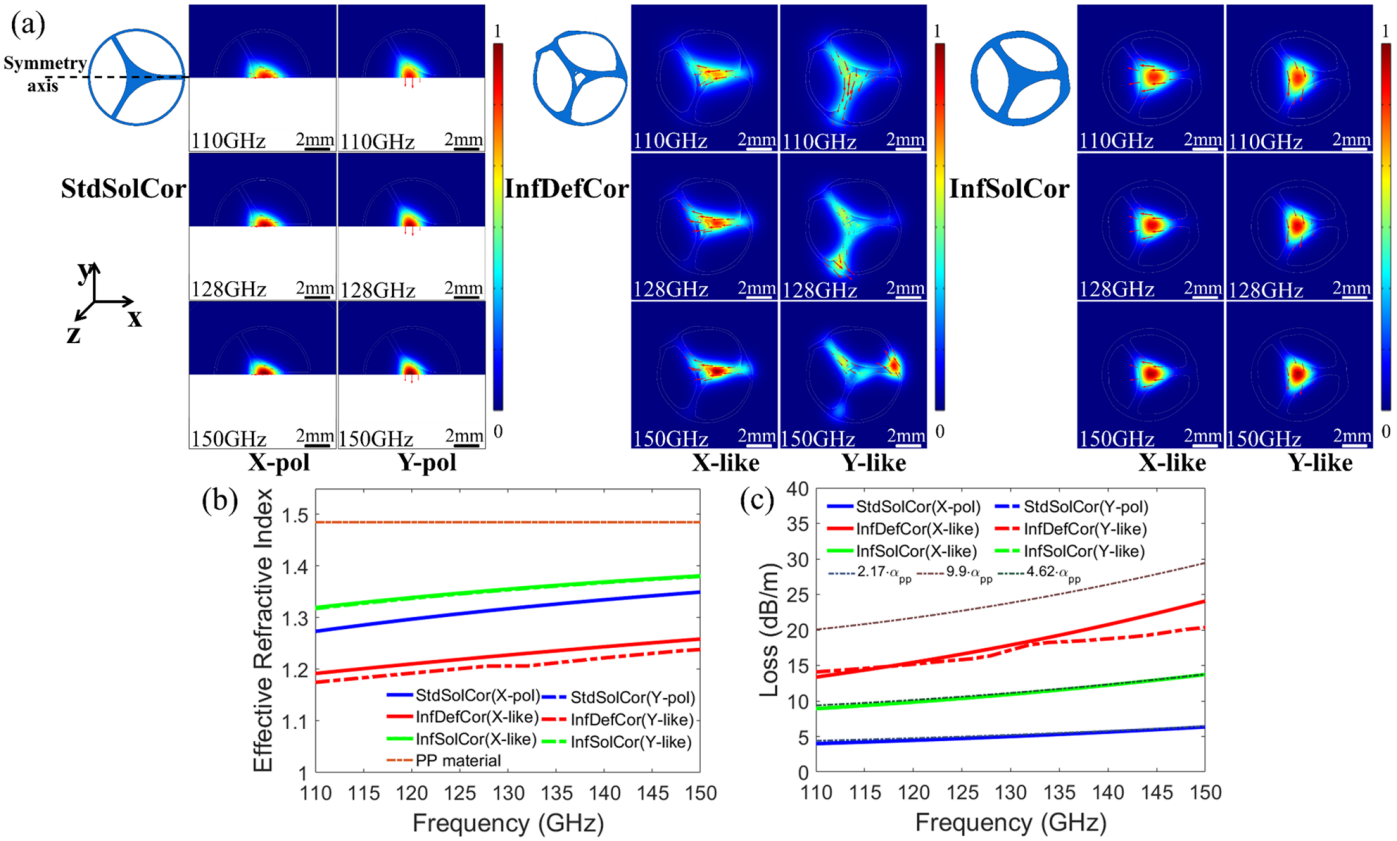
(**a**) Normalized electric field distribution and electric field direction (red arrows) of the two lowest order modes for the three fibers as a function of frequency. (**b**) Theoretical effective refractive indexes and (**c**) transmission losses (by power) for the two lowest order modes of three fibers as a function of frequency. Dotted lines show corresponding effective fiber material losses.

First, we note that the ideal fiber shown in Fig. 1 supports truly doubly degenerate fundamental modes, while experimental fibers shown in Figs. 2h and 3 support the nearly double degenerate fundamental modes. Generally, a certain linear combination of the degenerate or near degenerate modes will be excited at the fiber input depending on the excitation conditions. For practical applications, however, it is beneficial to optimize coupling conditions to preferentially excite a single mode to mitigate the negative effects of the inter-modal interference and inter-modal dispersion that can affect fiber information transmission (see Supplementary Materials Section S5 for a detailed explanation of the near degeneracy in experimental fibers). Before we address optimization of the modal excitation efficiency, we first study the modal structure for the three abovementioned fibers in the frequency range of 110–150 GHz using geometries shown in Fig. 3a. The normalized electric field distributions and electric field directions (red arrows) for the two lowest order modes for the three fibers are presented in Fig. 3a. Only in the case of StdSolCor, the X and Y polarizations can be unambiguously defined using reflection symmetries. Particularly, in the left panel of Fig. 3a we show field distributions of the properly symmetrized doubly degenerate fiber modes for StdSolCor, where X-polarized mode is calculated using half computational-cell and the Perfect Magnetic Conductor (PMC) boundary, while the Perfect Electric Conductor (PEC) boundary is used for calculating the Y-polarized mode. In the case of InfDefCor and InfSolCor, the two lowest order non-degenerate modes are calculated using a full computational cell, and their field distributions are presented in the middle and right panels of Fig. 3a. In what follows, we classify the lowest order modes of InfDefCor and InfSolCor as X-like and Y-like by analogy with the modes of a StdSolCor after inspection of their corresponding electric field distributions.

The effective refractive indexes of the two lowest order modes for the three fibers are presented in Fig. 3b and all show monotonic increase at higher frequencies. This is due to higher modal confinement in the waveguide core at higher frequencies as seen from the modal field distributions shown in Fig. 3a. Moreover, at any given frequency the InfSolCor has the highest effective refractive index as it features the largest core size. In contrast, the InfDefCor fiber has the smallest effective refractive index due to much weaker modal confinement caused by the air hole in the core center.

In Fig. 3c we present the calculated modal transmission losses, as well as adjusted bulk losses of the fiber material as a function of frequency. When calculating fiber losses we assume a frequency-dependent fiber material loss proportional to that of a bulk Polypropylene (from our prior study^14^) up to a multiplicative factor, which we choose to best reproduce the experimentally measured fiber transmission losses (see Characterization 2). In fact, there is no physical motivation for this particular form of the fitting function, which is rather chosen for convenience of presentation. Our motivation is to merely point out the reader’s attention to the fact that 3D printed fibers feature losses that can be significantly higher than those caused by just the material losses of a bulk Polypropylene. The additional fiber material losses are due to various surface and bulk defects introduced during 3D printing, while further analysis is needed to qualify and quantify the impact of such imperfections on the fiber optical properties (see Supplemental Materials Section S4 for more details). Thus, for StdSolCor, for the fiber core material loss (by power) we used $\sim 2.17\cdot {\alpha }_{PP}$, for the InfDefCor fiber we used fiber material loss of $\sim 9.9\cdot {\alpha }_{PP}$, and for the InfSolCor we used $\sim 4.62\cdot {\alpha }_{PP}$. The multiplicative factors were found using the least-squares method to minimize the fitting error between the experimental and numerical loss data. The fact that the two infinity-printed fibers have much higher fiber material losses than the bulk PP material is attributed to high scattering loss caused by the roughness of various fiber surfaces due to the printing process (see Fig. S2 in Supplementary Materials Section S3). Also, we note that standard FDM printing is highly optimized and, thus, results in the smallest scattering loss among all the fibers, while further work is in order to optimize the infinite-FDM process to further reduce scattering loss due to the manufacturing process.

For StdSolCor and InfSolCor, the two lowest order modes feature losses that are close to the effective fiber material losses since both modes are well confined inside the fiber solid core. In contrast, for InfDefCor, both lowest order modes are strongly present in the air holes inside and surrounding the fiber core, thus the modal losses are much smaller than the effective fiber material losses. Specifically, losses (by power) of the two almost degenerate lowest order modes for the StdSolCor in the range of 110–150 GHz are found to be 3.99–6.34 dB/m, with the corresponding value at the 128 GHz carrier frequency being 4.88 dB/m. For InfDefCor and InfSolCor, losses of the X-like modes in the range of 110–150 GHz are 13.36–24.06 dB/m for InfDefCor, and 8.93–13.72 dB/m for InfSolCor, with 17.35 dB/m (InfDefCor) and 10.7 dB/m (InfSolCor) losses at 128 GHz. Similarly, losses of the Y-like modes for the two fibers in the 110–150 GHz frequency range are 14.08–20.34 dB/m (InfDefCor) and 8.9–13.71 dB/m (InfSolCor), with the corresponding values at 128 GHz of 16.38 dB/m (InfDefCor) and 10.68 dB/m (InfSolCor). Furthermore, we also studied bending losses of the X-polarized or X-like modes of three bent fibers for two orthogonal fiber bending directions at 128 GHz, which shows that all the three fibers can readily tolerate tight bends with radius as small as 3 cm, resulting only in small loss increase of < 0.01 dB per 90° bend (see Supplementary Materials Section S6 for numerical analysis of fiber bending losses).

#### Study 2: excitation efficiency of the fiber modes

Here we study excitation efficiencies of the fiber modes using WR-6 waveguide flange as a source. The flange supports a single linearly polarized mode (along the X direction in the experiments) with a transverse electric field directed along the shorter side of a rectangular metallic waveguide. The complex modal excitation coefficient can be estimated using a well known expression^48^:

1 $$\begin{array}{c}{C}_{m}=\frac{\iint \left({E}_{mode}^{*}\times {H}_{wg}+{E}_{wg}\times {H}_{mode}^{*}\right)dxdy}{\sqrt{\iint 2Re\left({E}_{wg}^{*}\times {H}_{wg}\right)dxdy}\sqrt{\iint 2Re\left({E}_{mode}^{*}\times {H}_{mode}\right)dxdy}}\end{array}$$

where ${E}_{mode}$ and ${H}_{mode}$ are the transverse electric and magnetic fields of a given fiber mode, while ${E}_{wg}$ and ${H}_{wg}$ are the transverse electric and magnetic fields of the WR-6 waveguide flange. Then, relative power excited in the waveguide mode is given by ${{|C}_{m}|}^{2}$ which we refer to as excitation efficiency in the rest of the paper.

**Figure 4:**
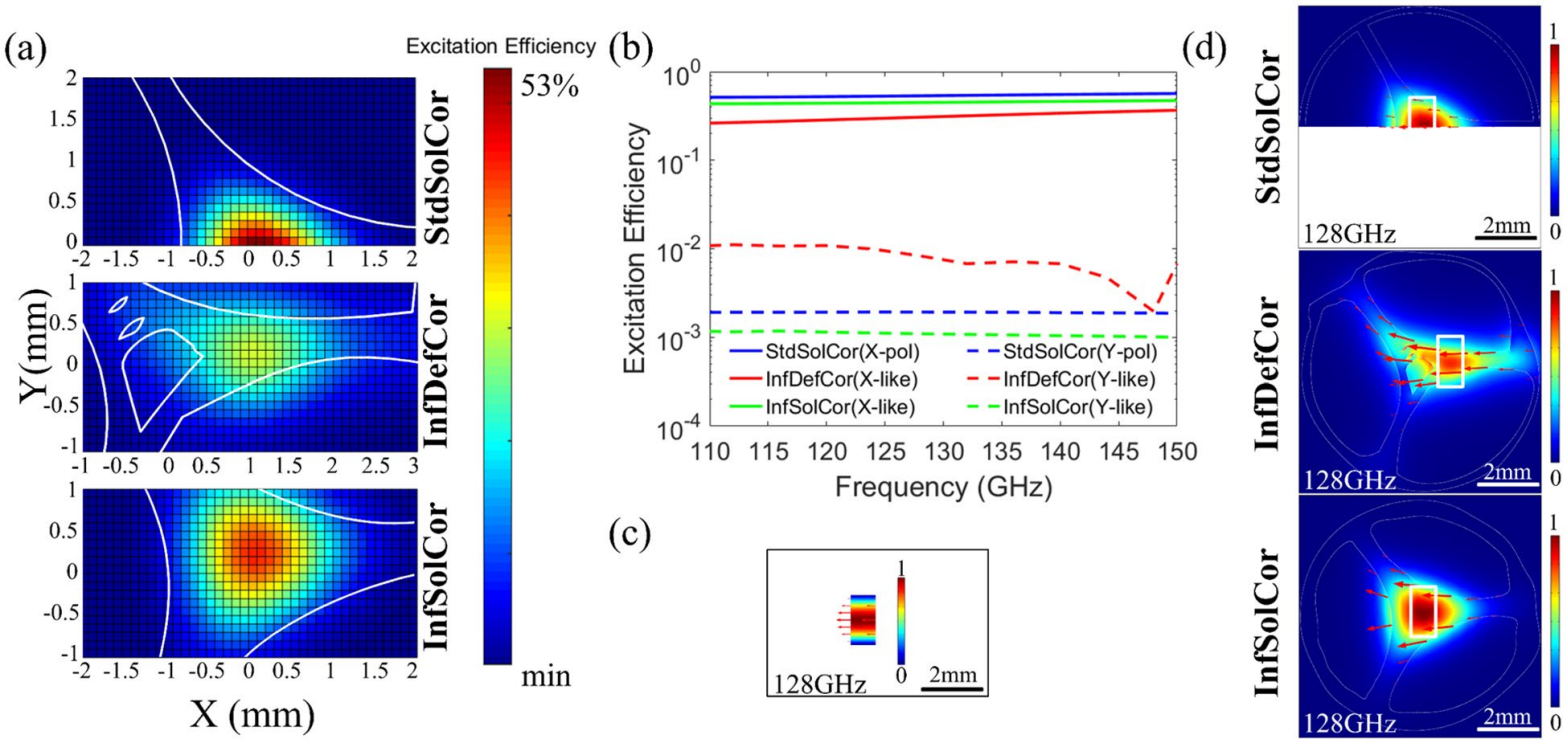
(**a**) Excitation efficiencies for the X-polarized and X-like modes for StdSolCor (top), InfDefCor (middle), and InfSolCor (bottom) as a function of the center position of the WR-6 waveguide flange at 128 GHz. WR-6 mode is X-polarized. Shown excitation efficiencies are optimized with respect to the fiber rotations. The white solid lines are fiber boundaries. (**b**) Excitation efficiencies of the two lowest order modes of the three fibers versus frequency for optimized coupling arrangement. Coupling is optimized for X-polarized or X-like modes at the carrier frequency of 128 GHz. Both fiber rotation and fiber position are optimized relative to a fixed WR-6 waveguide flange; Normalized electric field distributions and electric field directions (red arrows) for (**c**) the X-polarized mode of the WR-6 waveguide flange at 128 GHz and (**d**) the X-polarized or X-like modes of the three fibers at 128 GHz. White rectangles show optimized positions of the WR-6 waveguide.

To maximize the excitation efficiencies of the X-like modes for the InfDefCor and InfSolCor, one has to optimize both the fiber inclination and the relative position between the fiber and WR-6 waveguide. For the X-polarized StdSolCor mode, one can forgo the fiber rotation optimization step and simply choose polarization of the WR-6 waveguide mode along the fiber reflection symmetry axis (see Supplementary Materials Section S5 for details of the selective excitation optimization procedure). The map of excitation efficiencies for the X-polarized mode and X-like modes for the three fibers as a function of the center position of the WR-6 waveguide flange operating at 128 GHz is shown in Fig. 4a. These excitation efficiencies are also optimized with respect to the fiber rotations, assuming that polarization of the WR-6 waveguide mode [shown in Fig. 4c] is fixed along the X direction. The maximal excitation efficiencies of the X-polarized and X-like modes at the carrier frequency of 128 GHz for the StdSolCor mode reaches ~ 53%, while for the InfDefCor and InfSolCor they reach ~ 31% and ~ 45% respectively. The corresponding optimal fiber orientations and positions with respect to the WR-6 waveguide flange (indicated as white rectangles) are shown in Fig. 4d along with electric field distributions of the X-polarized and X-like modes. Thus optimized coupling efficiencies for the excitation of X-polarized and X-like modes over the frequency range of 110–150 GHz are shown in Fig. 4b, with excitation efficiencies for the StdSolCor ranging between ~ 51%-57%, and those for the InfDefCor and InfSolCor ranging between ~ 26–37% and 44–47%, respectively. Finally, in order to quantify the efficiency of a single mode excitation using the optimized procedure described earlier, in Fig. 4b we also present excitation efficiencies for the lowest-order Y-polarized and Y-like modes calculated for the optimal excitation conditions of the X-polarized and X-like modes, and note that they are all below 1%. This confirms that the optimized excitation procedure developed in our work guarantees an effectively single mode excitation with 14–27 dB suppression (by power) of other lowest order modes in all three fibers.

#### Study 3: modal group velocity dispersion and maximum bit rate

**Figure 5:**
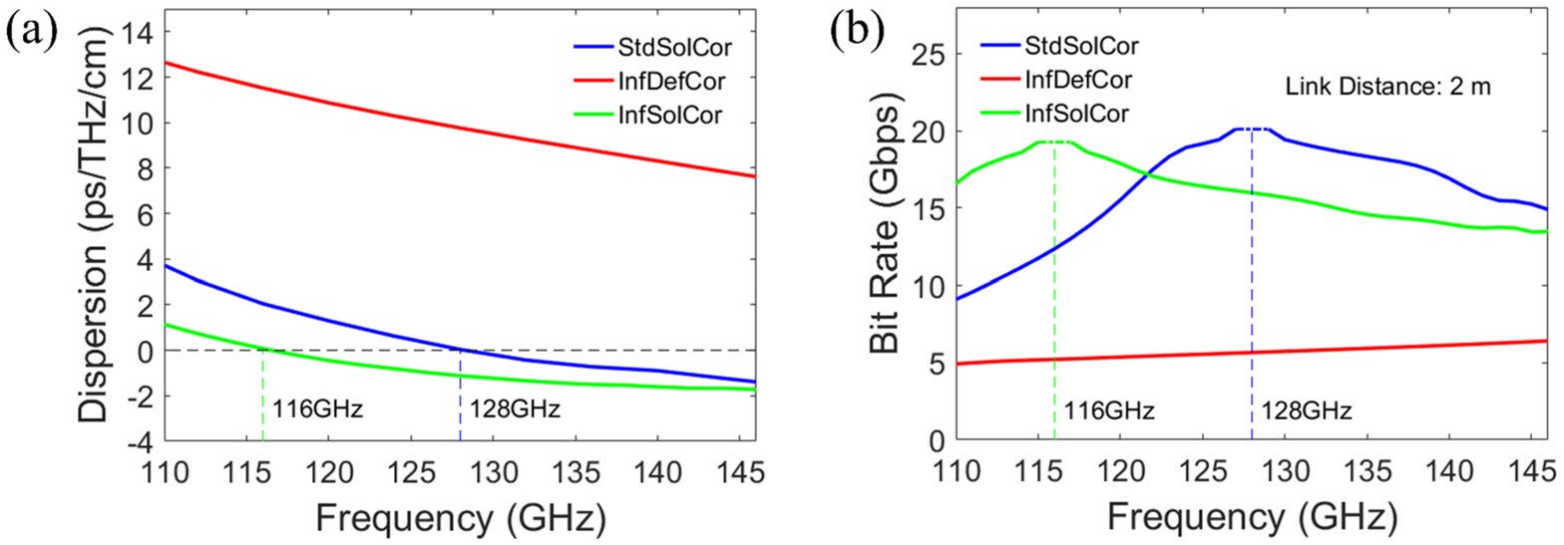
(**a**) Second-order dispersion of the X-polarized and X-like modes versus frequency for the three fibers. (**b**) Maximum bit rate supported by the three fibers for a link length of 2 m.

Another important factor affecting signal quality transmitted through optical fibers is dispersion. While the intensity of a received signal should be significantly above the receiver detection level, it is also essential to minimize signal distortion due to dispersion. The dispersions of X-polarized or X-like modes for the three fibers as a function of frequency are shown in Fig. 5a. The dispersion of StdSolCor at the carrier frequency of 128 GHz is near zero by design while it is in the range of -2 to 4 ps/THz/cm in the whole frequency range. For InfSolCor, the dispersion curve is similar to that of a StdSolCor with the zero-dispersion frequency somewhat shifted to 116 GHz due to deviation of the fiber geometry from the optimal one during manufacturing. Finally, dispersion of the InfDefCor is large and positive ~10 ps/THz/cm in the whole frequency range due to a hole defect in the fiber core. Assuming an infinite Signal to Noise Ratio (zero noise), the maximal bit rate supported by a single mode fiber at a given carrier frequency for a simple ON-OFF keying modulation can be estimated as^49^:

2 $$\begin{array}{c}{BR}_{max}=1/\left(4\sqrt{\left|{\beta }^{\mathrm{^{\prime}}\mathrm{^{\prime}}}\right|L}\right)\end{array}$$

where ${\beta }^{{^{\prime\prime}}}$ is the second order derivative of the modal propagation constant, and $L$ is the fiber length. At the frequency of zero dispersion the maximum error-free bit rate can be estimated using the third order modal dispersion^49^:

3 $$\begin{array}{c}{BR}_{ZD}=0.324/\sqrt[3]{\left|{\beta }^{\mathrm{^{\prime}}\mathrm{^{\prime}}\mathrm{^{\prime}}}\right|L}\end{array}$$

where ${\beta }^{{^{\prime\prime\prime}}}$ is the third order derivative of the modal propagation constant. In Fig. 5b we plot the maximal estimated bitrate ${BR}_{max}$ as a function of the carrier frequency, while capping its maximal value by ${BR}_{ZD}$, while assuming a $L=2 m$ fiber length. From Fig. 5b we see that in the vicinity of the corresponding zero dispersion frequencies, the 2 m-long StdSolCor and InfSolCor can support error-free transmission bit rates over 20 Gbps (at 128 GHz) and 19 Gbps (at 116 GHz). Meanwhile, at 128 GHz the 2 m-long InfDefCor and InfSolCor can support the error-free transmission rates of up to ~ 5 Gbps and ~ 16 Gbps, which are equally attractive for various short-range fiber-assisted communication applications.

### Experimental characterization of the THz fiber communication links

The experimental characterizations were carried out using an in-house photonics-based THz communication system detailed in our prior study^14^. The same system can be used both in the Continuous Wave (CW) THz spectroscopy and THz communication mode by simply disabling or activating the communication unit (see Supplementary Materials Sections S7 and S9 for details of the two systems).

#### Characterization 1: mode field imaging

**Figure 6:**
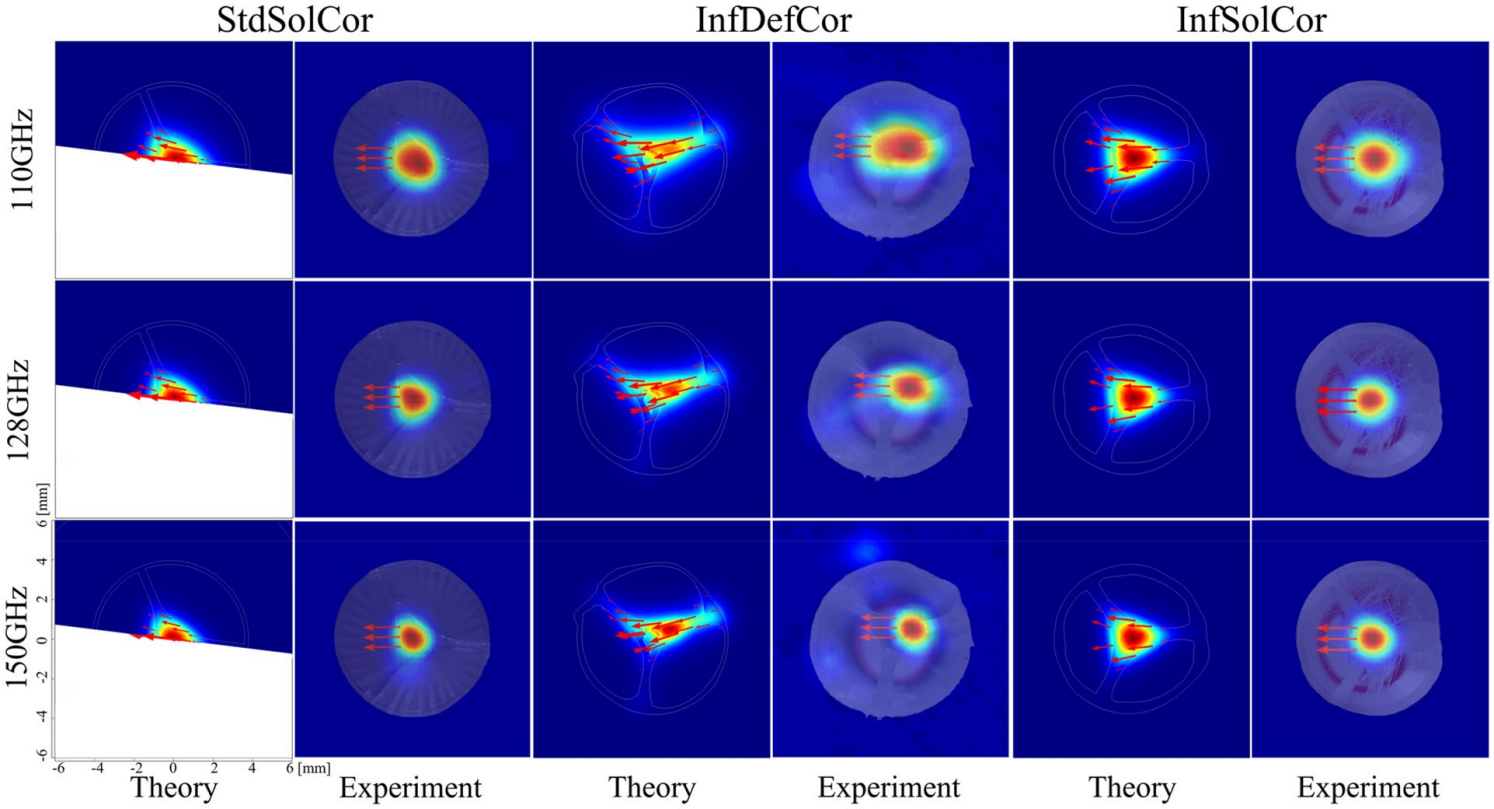
Comparison of the theoretically and experimentally measured electric field distributions of the X-polarized and X-like modes of the StdSolCor (0.25 m), InfDefCor (0.75 m), and InfSolCor (1.6 m). Electric field directions are shown as red arrows. In experimental measurements, electric field direction is defined by the orientation of the WR-6 waveguide flange, which is horizontal.

To verify the single-mode guidance of the fabricated 3D printed fibers, a near-field THz modal imaging has been carried out at the output end of the fiber using the CW THz spectroscopy system (see Supplementary Materials Section S7). The experimental and theoretical modal field distributions of the 3D printed fibers at three different frequencies, along with the electric field directions (red arrows), are presented in Fig. 6. The red arrows shown in the theoretical result are the electric field directions of the X-polarized or X-like modes guided in the three fibers, while arrows in the experimental figures show the electric field direction in the fundamental mode of the WR-6 waveguide flange used for waveguide excitation at 128 GHz. It should be noted again that the polarization directions of the transmitter and receiver antennas were always fixed to the horizontal direction, while theoretical field distributions in Fig. 6 are rotated to match the fiber orientation of the experimental setup. Overall, there is a good correspondence between the theoretical and experimental modal images, while minor differences come from the fact that our THz imaging setup only measures a single horizontal field component of the electric field while averaging it over ~1mm aperture. Thus, one clearly observes stronger modal confinement in the core at higher frequencies for all three fibers, as well as field displacement into the bridge region due to hole defect in the InfDefCor core.

#### Characterization 2: transmission loss measurements

**Figure 7:**
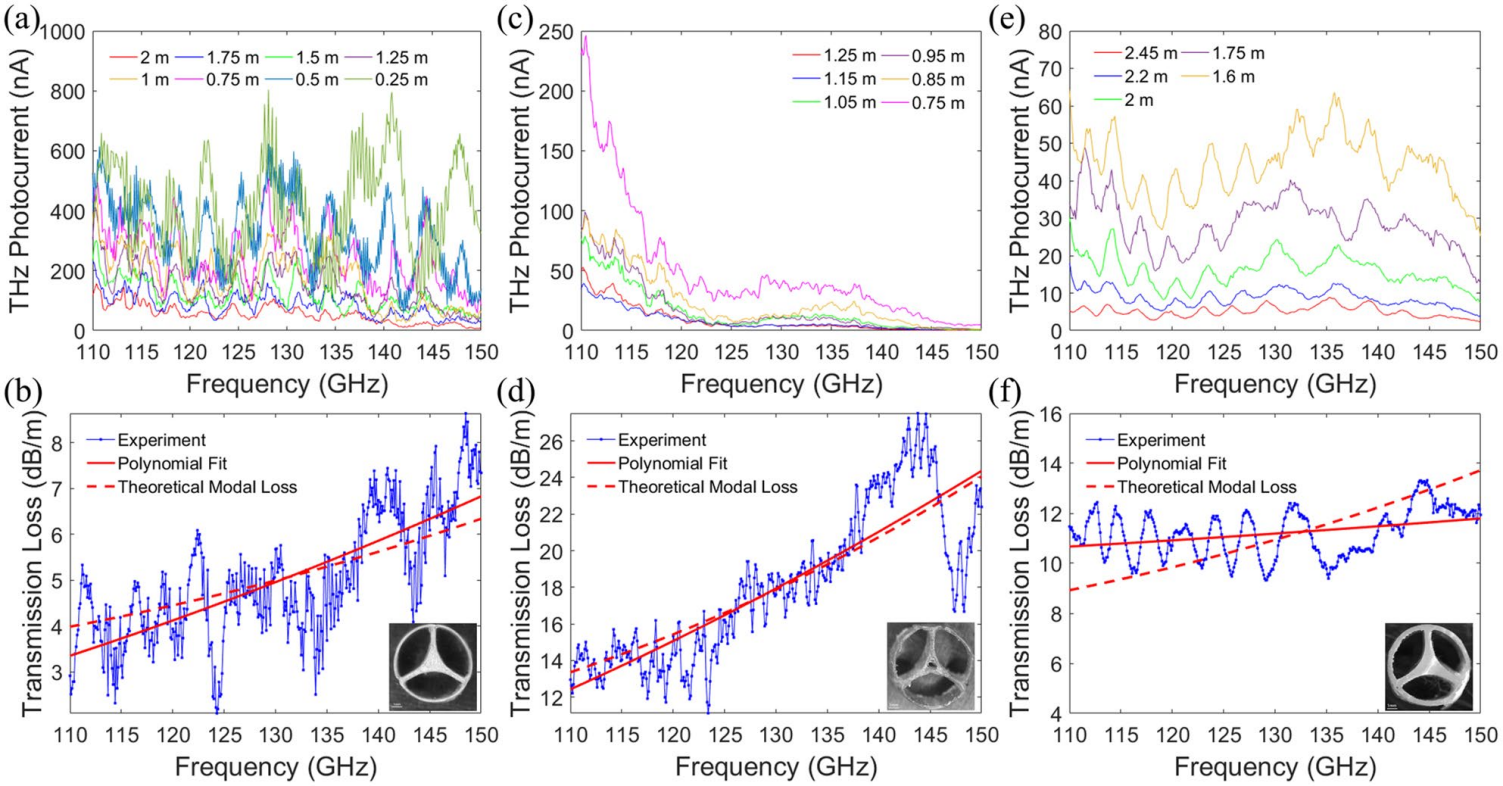
Experimental transmission spectra and losses (by power) of the X-polarized and X-like modes for (**a**, **b**) StdSolCor, (**c**, **d**) InfDefCor and (**e**, **f**) InfSolCor. Inset: microscope images of the fiber cross-section.

Firstly, the transmission losses of the 3D printed fibers presented above were measured using the standard cut-back method. The measurements were carried out with the CW THz spectroscopy system (see Supplementary Materials Sections S7 and S8 for details). A total of 8, 6, and 5 transmission spectra in the range of 110–150 GHz were obtained respectively for the StdSolCor, InfDefCor, and InfSolCor fibers (see Fig. 7a, c, e). The maximal length of each fiber link was mostly limited by the fiber losses at all frequencies (see Characterization 3 for details). At a given frequency $\nu$, fiber loss $\alpha \left(\nu \right)$ is estimated by minimizing the least squares deviation of the experimentally measured transmitted intensities $I$ (measured as photocurrents) as a function of the fiber length from the theoretically expected one (4). In this fitting procedure, the frequency dependence of the fiber loss is assumed to be second order polynomial:

4 $$\begin{array}{c}I={I}_{0}\cdot exp\left(-\alpha \left(\nu \right)L\right)\\ \alpha \left(\nu \right)={a}_{2}{\nu }^{2}+{a}_{1}\nu +{a}_{0}\end{array}$$

Thus found fiber loss is shown in Fig. 7b, d, f as solid red curves. Similarly, the transmission losses of the X-polarized (StdSolCor) and X-like (InfDefCor and InfSolCor) modes are numerically calculated using COMSOL mode solver and are shown as dashed red curves on the same figures, and a good agreement with the measurements is observed. At the carrier frequency of 128 GHz, the measured fiber transmission losses (by power) are found to be 4.79 dB/m, 17.34 dB/m, and 11.13 dB/m for the StdSolCor, InfDefCor, and InfSolCor correspondently.

#### Characterization 3: bit error rate measurements

Next, the bit error rate (BER) measurement was carried out to evaluate the communication performance of the 3D printed fibers using the photonics-based THz communication system. The communication unit was enabled and the 3D printed fibers were coupled to the transmitter and receiver antenna in a similar arrangement to the modal loss measurement (see Supplementary Materials Section S9).

**Figure 8:**
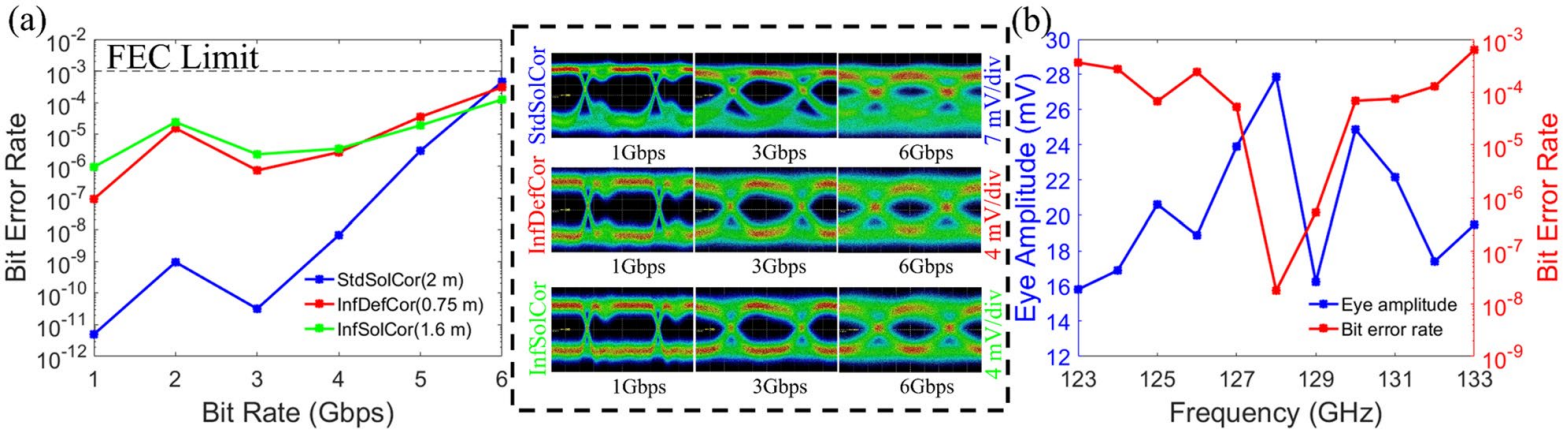
(**a**) Measured bit error rate versus bit rate for StdSolCor (2 m), InfDefCor (0.75 m), and InfSolCor (1.6 m) at the carrier frequency of 128 GHz. Inset: The recorded eye pattern for different bit rates. (**b**) Measured eye amplitude and bit error rate for StdSolCor (2 m) at the bit rate of 4 Gbps as a function of frequency.

The StdSolCor was assembled from 3D printed sections of 25 cm each to the total length of 2 m, while longer fiber links can be readily achieved by connecting more sections. The maximal fiber lengths of InfDefCor and InfSolCor for conducting the BER measurement were limited to 0.75 m (InfDefCor) and 1.6 m (InfSolCor). The fiber lengths were chosen to result in similar total fiber link transmission losses of ~ 15–16 dB (which includes both coupling and fiber losses) as measured by the eye amplitudes of the oscilloscope. As the noise level of our THz communication system is − 34 dBm (~ 2.5 mV) and the signal strength is −  6.6 dBm, then, after fiber transmission we are still operating ~ 12 dB in power above the noise level. The BER and corresponding eye patterns at 128 GHz for the fibers at the bit rates of 1–6 Gbps are presented in Fig. 8a.

From Fig. 8a, we see that the lowest bit error rates (BER < 10^–8^) were observed for the StdSolCor, particularly for the bit rates below 4 Gbps, due to fiber operation near the frequency of zero modal dispersion. Although the fiber length is the longest among the three fibers (2 m), the eye amplitude measured for the received signal is larger than those for the InfDefCor and InfSolCor because of the StdSolCor lower transmission losses. We also note that, in the case of StdSolCor fiber, the bit error increases rapidly for higher bit rates. The reasons for such a rapid deterioration of the BER involve multiple factors which require further investigation (see Supplementary Material Section S9 for details). One reason is that for higher bit rates, the channel bandwidth increases, and the fiber can no longer be considered as operating at the frequency of zero dispersion. In optical communications, one frequently uses dispersion flattened fiber designs to increase the bandwidth of low dispersion operation and a similar tactic can be used for the advanced design of THz fibers. It is worth mentioning that, an error-free transmission (BER < 10^–12^) was achieved at the bit rate up to 5.2 Gbps with StdSolCor of 1.5 m-length. In comparison, for the InfDefCor and InfSolCor, the BER is much higher than for StdSolCor as they operate away from their respective zero dispersion frequencies. At the same time, for all three fibers, signal transmission with BERs below the forward error correction (FEC) limit (10^–3^) is supported even at bit rates as high as 6 Gbps. To study the effect of the choice of a carrier frequency on the fiber performance, we conducted BER measurements in the vicinity of a zero dispersion frequency of the StdSolCor fiber and recorded the eye amplitudes for the 2 m-long StdSolCor fiber at the bit rate of 4 Gbps by varying the carrier frequency from 123 to 133 GHz as shown in Fig. 8b. From this figure, we observe that by varying the carrier frequency, the eye amplitude (defined as the min to max value of a signal) varies from ~ 16 to ~ 28 mV, while achieving its maximal value at 128 GHz. While the eye amplitude drops suddenly at 129 GHz (which is solely due to the response of our detector as established earlier^7^), nevertheless, the bit error rate (~ 10^–8^) shows a clear minimum in the whole 127–130 GHz range. While this behavior can be attributed to operation near zero dispersion frequency of 128 GHz as predicted theoretically in Fig. 5, one has to be cautious due to the potential contribution to the signal degradation of a resonant cavity of a photomixer at the fiber coupling end (see Supplementary Material S8 for details).

In conclusion, we project that by resorting to dispersion flattened designs in the vicinity of zero dispersion frequency, and by optimizing the 3D printing quality, transmission rates of ~ 10–20 Gbps in the 3D printed ~ 10 m-long THz fiber links are possible even when using low-power (~ 0.1 mW) THz optical sources.

## Conclusion

In this work, we explored an infinity 3D printing technique to fabricate continuous several-meter-long low-loss near-zero dispersion suspended-core polypropylene fibers for application in terahertz communications. The suspended-core geometry was chosen to shield the mode from external influence and simplify the handling of such fibers in practical applications. While Polypropylene polymer was chosen as one of the lowest loss plastics in the THz regime, in fact, it is rarely used with the FDM technique due to the heavy warping of the material during printing. Therefore, particular attention was paid to process parameter optimization for printing with low-loss polypropylene plastic, as well as in-depth comparison between three fibers printed using standard and infinity 3D printers.

Experimentally, the transmission losses (by power) of the three fibers produced using standard and infinity FDM techniques were measured to be 4.79 dB/m, 17.34 dB/m, and 11.13 dB/m for the StdSolCor, InfDefCor, and InfSolCor respectively at the carrier frequency of 128 GHz. Subsequently, the BER measurements were carried out by varying the bit rate between 1 and 6 Gbps for the three fibers. Signal transmission with BER far below the FEC limit was observed for the 2 m-long StdSolCor, 0.75 m-long InfDefCor, and 1.6 m-long InfSolCor, respectively. Additionally, the BER measurements were conducted by varying the carrier frequency between 123 and 133 GHz for the 2 m-long StdSolCor at the bit rate of 4 Gbps, and a clear minimum in the BER (~ 10^–8^) was observed at 128 GHz, which is a zero dispersion frequency chosen for the StdSolCor design. Moreover, an error-free transmission was achieved for the bit rate up to 5.2 Gbps using StdSolCor with a length of 1.5 m which can already be interesting for practical applications.

Finally, the near-field imaging of the fiber fields was performed by raster scanning of the fiber output facets with a sub-wavelength aperture. It showed strong modal confinement in the suspended-core region well inside of the outer protective shell.

We believe that our work has demonstrated that infinity 3D printing holds strong potential for the development of THz fibers and fiber components via single-step fabrication of unlimited-length fibers featuring complex geometrical cross-sections while using low-loss plastics.

## Supplementary Information

Supplementary Information 1.

Supplementary Video 1.
