# Supplementary material for: Infinity additive manufacturing of continuous microstructured fiber links for THz communications
Source: Sci Rep. 2022 Mar 16;12:4551. doi: 10.1038/s41598-022-08334-6 (PMC8927297; doi:10.1038/s41598-022-08334-6)
Supplement: Supplementary file 1 — Supplementary Information 1. [file 41598_2022_8334_MOESM1_ESM.docx]

**Infinity Additive Manufacturing of Continuous Microstructured Fiber Links for THz Communications: Supplementary Materials**

# GUOFU XU+, KATHIRVEL NALLAPPAN+, YANG CAO, AND MAKSIM SKOROBOGATIY*

École Polytechnique de Montréal, Department of Engineering Physics, Montréal, H3T 1J4, Canada

*[maksim.skorobogatiy@polymtl.ca](mailto:maksim.skorobogatiy@polymtl.ca)

+these authors contributed equally to this work

# Section S1: Motivation for using fiber-based THz links

Note 1: In fact, at lower THz carrier frequencies (~100-150 GHz), the free-space links can be as long as several km^1-4^, while at higher THz frequencies (200-300 GHz) they are reduced to ~10-20 meters^5,6^ due to a more pronounced atmospheric absorption^7-10^. In both cases, however, THz communication links have to be designed to provide a safety power margin to accommodate for the potential increase in atmospheric attenuation, which becomes especially critical for longer link lengths. While the THz waves are certainly better in rainy or foggy environments than the visible or infra-red light as Mie scattering at THz frequencies is significantly less pronounced, however, the atmospheric absorption in severe weather conditions (heavy rain, fog, snow, etc.) is still a problem that needs to be considered when developing a free-space THz communication system^11-13^. Particularly, scattering on hydrometeors still occurs in the rain, fog, and clouds, and it can reach up to 100 dB/km depending on the precipitation rate (especially for frequencies higher than ~1 THz)^14,15^.

Note 2: Highly directive THz antennas could have a higher gain than the microwave antennas, therefore, Frii’s loss for THz antennas could be much smaller than the corresponding loss for the microwave antennas of the same size. That said, in the case of long free-space THz links with distances on the order of 100m-1km ^16,17^, or when using relatively small receivers and detectors, beam diffraction cannot be ignored. In the case of an optics-based system that we use in our work, with the photomixer-based emitter and detector (aperture sizes of ~2 cm diameter), after $L$=10 m propagation of 150 GHz carrier wave ($\lambda$= 2 mm), the Frii’s loss due to beam diffraction^18^ will be high:

$$\begin{aligned} Loss=-10\cdot{log}_{10}\left( \frac{P^{r}}{P^{t}} \right)=-10\cdot{log}_{10}\left( \frac{A^{r}A^{t}}{\lambda^{2}L^{2}} \right)=36 dB\#\left( S1 \right) \end{aligned}$$

where $P^{t}$and $P^{r}$ are the transmitter power and received power, $A^{t}$and $A^{r}$are the effective areas of transmitting and receiving antennas.

Note 3: While THz antennas are considerably more directive than the microwave ones of the same size (due to the much smaller wavelength of the THz waves), this, however, might be a disadvantage for the free-space THz links in geometrically complex environments. Although THz beam steering or beamforming with phased array antennas may be useful, these are still in the early stage of development. For the indoor THz communications, in particular, one would have to rely on electronically steerable phased array antennas or make use of multiple reflections from the indoor walls/floor/ceiling, to allow for an automatic connection between transmitter and receiver, however, the required smart antennas at THz frequencies are not yet available ^8,14,15^. In this respect, augmenting THz wireless systems with the THz fiber links can offer reliable system performance in the geometrically complex communication environments (ex. a multistory building) to provide reliable points of THz wireless access within smaller and less complex communication environments (such as individual offices), while using a THz fiber connection between such access points (ex. through the building walls).

# Section S2: Theoretical Fiber Structure Optimization

The optimization of the fiber structure was conducted using finite element COMSOL Multiphysics software. Half computational cell with Perfect Magnetic Conductor (PMC) boundary conditions set along the symmetry reflection plane was used to study the lowest order mode featuring an electric field directed preferentially along the plane. The effective refractive index and absorption coefficient (by power) of the Polypropylene polymer were fixed at 1.49 and 0.02 cm^-1^ (~8.69 dB/m). The fiber outer cladding was first assumed to be infinite plastic to quantify core to cladding radiation (leakage) loss due to finite bridge thickness $H_{br}$ and length $L_{br}$. The radius ${R1}_{cr}$ of the three air holes was defined as ${R1}_{cr}={R2}_{cr}\cdot sin\left( \pi/3 \right)-{H_{br}}/2$. The distance ${R2}_{cr}$was varied between 2.5-6.0 mm with a step value of 0.1 mm, to realize fibers of core size $2\cdot({R1}_{cr}-{R2}_{cr})$ ranging from ~0.87 mm to ~2 mm, and tune the spectral position of the zero dispersion frequency. The bridge length $L_{br}$ that controls coupling of the core and cladding regions was varied between 2.0-4.5 mm with a step value of 0.5 mm. The values for $H_{br}$ were set as 0.2 mm or 0.4 mm due to practical limitations when fabricating using 3D printing and to provide sufficient mechanical strength for the suspended core with minimal deformations. Therefore, by varying ${R2}_{cr}$ while fixing the values for $H_{br}$ and $L_{br}$, the fiber design featuring near zero dispersion at 128 GHz can be obtained. By comparing these dispersion optimized fibers featuring different parameter combinations of $H_{br}$ and $L_{br}$, we then found that the total fiber losses are comparable for the fibers with $L_{br}$= 4.5 mm-4 mm, while increasing rapidly (by a factor of 2-3) when using shorter bridge lengths $L_{br}$= 3.5 mm-2 mm. Therefore, the optimal $L_{br}$ was set to 4 mm to minimize the fiber core to cladding leakage loss, while still keeping the outer fiber size as small as possible to maintain the fiber flexibility. Furthermore, for the fibers featuring $L_{br}$= 4 mm and $H_{br}$= 0.4 mm or 0.2 mm, we found that while the one with $H_{br}$= 0.2 mm has a marginally smaller loss, however consistent printing with such a thin layer of internal fiber microstructure was problematic. Thus, the optimal $H_{br}$ was identified as 0.4 mm, with the corresponding values of ${R2}_{cr}$= 4.5 mm and ${R1}_{cr}$= ~3.7 mm. The theoretical radiation leakage loss of thus optimally designed fiber with an infinite cladding is ~0.05 dB/m at 128 GHz, which can be also considered as an approximation to the core-to-clad leakage loss in the printed fiber with a finite cladding. Given that the Polypropylene absorption loss is ~2.4 dB/m at 128 GHz, we conclude that the core-to-clad leakage loss constitutes only a small fraction of the fiber material absorption loss. Finally, during manufacturing, the cladding thickness $H_{cl}$ was chosen as 0.2 mm as it has enough mechanical strength to work as a robust mechanical shell for the fiber while being practical to fabricate using 3D printing.

# Section S3: Optimization of the print quality using a standard FDM printer

FDM technique unavoidably results in surface roughness with sizes comparable to the deposited layer thickness. Additionally, when printing the fiber bulk regions with 100% filling by volume, accidental air trapping is possible. Both surface roughness and air trapping in the bulk lead to additional scattering loss. At the same time, when operating in the low THz frequency range (most suitable for communication applications) the wavelength size is typical ~1 mm, while the layer thickness of an FDM printer, as well as its transverse resolution, are in the ~0.1 mm range. Thus, the surface and bulk roughness are deeply subwavelength, and scattering from such defects should obey the Rayleigh law.

Therefore, as an indicator of the print quality, we use sample transparency in the visible. To achieve the best transparency (highest material uniformity) at 100% filling factor, dozens of cylindrical pellets (15 mm diameter and 4 mm height) were printed with somewhat different combinations of the printing parameters. In our studies, we used “Raise3D Pro2” FDM printer that features high positional precision (~1 um in the XY plane and ~10 µm along the Z-axis), as well as high resolution in both horizontal (nozzle size of 0.2 mm) and vertical (minimal layer thickness of 0.1 mm) directions. The 1.75 mm natural transparent PP filament was employed for printing since it has high transparency and low absorption loss in THz regime ^19^. A standard “Rectilinear” infill pattern is used in the optimization process as it usually results in mechanically isotropic prints in the build plane. The optimal extrusion temperature for the Polypropylene is reported in the 190-250 ℃ range^20^, and in our experiments is chosen to be 240 ℃ according to the filament manufacturer recommendations.

Next, we perform a multiparameter optimization of the printing process for the Polypropylene material.

The Pellets printed with different infill flow rates of 90%, 100%, 110%, and 120% at a constant infill speed of 30 mm/s and a fixed layer height of 0.15 mm are shown in Fig. S1(a). The optimal transparency is achieved at somewhat elevated infill flow rates of ~110%, as air gaps between the adjacent extruded lines tend to be minimized as an extra amount of material is squeezed out. However, when using higher flow rates, significant material overflow occurs leading to an increase in surface roughness. The pellets printed with different infill speeds of 10 mm/s, 30 mm/s, 120 mm/s, and 180 mm/s at a fixed flow rate of 110% and a fixed layer height of 0.15 mm are shown in Fig. S1(b). The best transparency is obtained at the relatively slow infill speeds of ~30 mm/s which tend to result in wider extruded lines and denser prints. However, if the infill speed is set too low, over-extrusion will occur ultimately increasing surface roughness and inhomogeneity of a print. Conversely, excessively high infill speeds will increase gaps between the adjacent lines, resulting in higher content of trapped air and poor transparency. Finally, in Fig. S1(c), we show pellets printed using layer heights of 0.13 mm, 0.15 mm, 0.17 mm, and 0.19 mm printed at a fixed infill rate of 110 % and infill speed of 30 mm/s. From this figure, we see that the layer height also makes a significant impact on the transparency of a print, with an optimal layer thickness being 0.15 mm. While thinner printing layers generally provide higher printing quality and mechanically stronger prints, however, at very small layer thicknesses, fluctuation in the flow rate of molten plastic can lead to significant variations in the layer thickness and, consequently, degradation of the print quality.


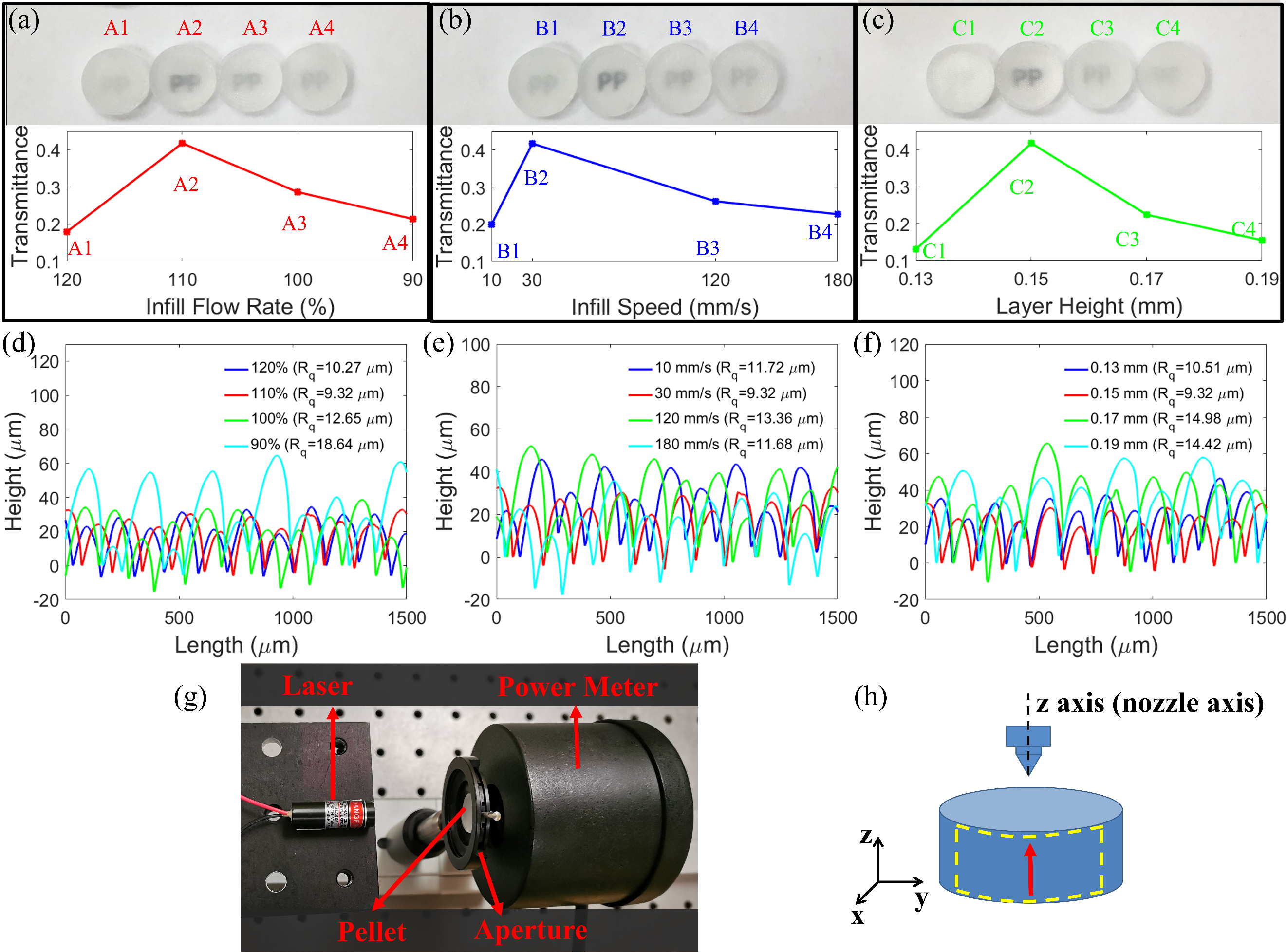


Figure S1. (a) Pellets printed with different infill flow rates of 120%, 110%, 100%, 90% (decreasing from left to right), and their corresponding normalized transmittances at 650nm. (b) Pellets printed with different infill speeds of 10 mm/s, 30 mm/s, 120 mm/s, and 180 mm/s (increasing from left to right), and their corresponding normalized transmittances at 650nm. (c) Pellets printed with different layer heights of 0.13 mm, 0.15 mm, 0.17 mm, and 0.19 mm (increasing from left to right), and their corresponding normalized transmittances at 650nm. Surface roughness distributions of the pellets printed with different (d) infill flow rates, (e) infill speeds, and (f) layer heights. (g) Experiment setup to measure the transmittance of the 3D printed pellets. (h) Schematic of the printed pellets with marked profiling surface (yellow dashed region) and profiling direction (red arrow).

Dependence of the pellet quality on printing parameters was also characterized by recording optical transmission of a 650 nm laser through the 3D printed samples [see Figs. S1(a-c)]. Particularly, an aperture (IDA25 from Thorlabs Inc) was used to fix the pellets and block the stray light, a laser (FU650AD5-BC10/BD10) was used to launch the beam through the pellet, while the intensity of the transmitted light was measured using a power meter (P/N 1Z01500 from Nova Display Systems, Inc) [see Fig. S1(g)]. Normalizing by the intensity of light going through an empty system we obtained transmittances that are presented in Figs. S1(a-c). From this data, we see clearly that the sample transparency, which is anticorrelated to the number of defects in the printed structure, can be optimized by varying printing parameters. In addition, several other parameters can influence the printing quality of certain structures, and, therefore, also need to be optimized. Thus, the combination of printing speed and infill overlap (with the walls) has a great impact on the size and quality of the printed slender parts, which is crucial for printing thin bridges in the fiber cross-section. Thinner shells and structures can be obtained when using higher printing speed and smaller infill overlap parameters. In addition, the heavy warping that often happens in the first few layers when printing with a PP filament can be addressed by using the appropriate first layer printing speed and built plate temperature.

Moreover, a preliminary study of the surface roughness for the pellets and exposed walls of the fiber cores was carried out using the Dektak 150 Surface Profiler (from Veeco, Inc). For pellets, the measurements were performed for the surfaces oriented along the vertical Z-axis [see schematic in Fig. S1(h)], which is also the printing direction for the StdSolCor fiber. Optimization of the printing parameters to minimize the pellet roughness along the vertical direction is meant to minimize the roughness of the core walls of a StdSolCor fiber. The root mean square average of the profile heights over the evaluation length also commonly referred to as the roughness R_q_ are shown in Figs. S1(d-f). Interestingly, we find that the pellets with the lowest sidewall roughness [red curves in Figs. S1(d-f)] correspond to the pellets featuring the highest transmittance [Figs. S1(a-c)]. Note that the anticorrelation between the extent of the sidewall roughness, and the sample transparency is somewhat nontrivial to rationalize and deserves further studies. Indeed, sample transparency is influenced strongly by the bulk defects in the structure of the pellet, which are not trivial to characterize using profilometry. At the same time, the established anticorrelation seems to suggest that pellet side roughness alone can be used to judge the bulk quality of the pellets.

Furthermore, in Figs. S2(c) and S2(d-f), we present some representative microscope images and surface roughness distributions along the core walls of the three fibers. As fiber cores have three concave walls, we present separate data for all the walls. All the measurements were conducted along the direction of material layer deposition [see blue arrows in Fig. S2(a) for StdSolCor fiber and blue arrows in Fig. S2(b) for InfDefCor and InfSolCor fibers]. We note that in the case of standard printing (StdSolCor), wall roughness distributions are similar to each other due to the isotropic printing process in the fiber cross-section. In contrast, for infinity printing (InfDefCor, InfSolCor), surface roughness distributions can be very different for the three core walls as printing is performed at 45° inclination with respect to the fiber length.

Next, we note that for the StdSolCor fiber fabricated using standard 3D printing, the average roughness R_q_=22.16 µm [Fig. S2(d)] is significantly higher than that for the pellet R_q_=9.3 µm [red curve in Fig. S1(d)] both of which were printed using the same processing parameters. This suggests that the fiber core surface roughness is influenced not only by the printing parameters but also by the geometrical structure of the printed fibers. Further studies are, therefore, needed to clarify this phenomenon which is beyond the scope of this paper.

Moreover, we find that the core wall roughness in the InfDefCore and InfSolCore fibers [see, for example, Figs. S2(e,f)] differ dramatically between the three core walls in the Rq=23-144 µm and Rq=17-57 µm ranges for the two fibers respectively. These results are found by inspecting 5 different fiber sections (each featuring 3 walls) located along the whole fiber length. A surprising finding is that one of the three core walls in the infinity printed fibers features rough patches separated every ~0.5-1 cm along the fiber length, while the other two walls remain relatively smooth along the whole fiber length. Roughness in those patches can be 3-6 times higher than the roughness of the other walls in the same fiber, while between the rough patches one observes similar roughness ~20-30 µm for all three walls. As a consequence, losses of the infinity core fibers ~10-20 dB/m are higher than those of a standard printed fiber ~5 dB/m (see Fig. 7). Therefore, we conclude that the nature of a higher transmission loss in the infinity-printed fibers seems to be related to the presence of rough patches along one of the three core walls in the infinity-printed fibers. At the same time, the reason for the appearance of those patches during fabrication, as well as their distribution along the fiber length deserves a separate study.

Additionally, we find that infinity fibers show somewhat stronger variations in the fiber cross-section geometry along the fiber length (compared to standard printed fibers) as surmised from the images of the fiber cross-sections taken at the beginning, middle and end of the ~2 m-long fiber links [see Figs. S2(g-i)]. The nature of these variations can be related to the anisotropic printing process and some difficult to control factors such as stresses at the interface between the fiber and the support belt of an infinity printer, while more studies are needed to confirm and quantify this phenomenon.

Finally, in Fig. S3 we present cross-sections and profilometry of the cleaved surfaces of the two fiber cores. This study is meant to probe bulk defects inside the fiber cores. The surfaces were prepared by freezing the fiber sections in liquid nitrogen and then snapping the fibers after making a shallow incision with a sharp blade. Remarkably, from the microscope images [Figs. S3(a-d)] we cannot observe any striation marks due to the layer-by-layer deposition process, with the fiber cores seemingly completely solidified, as confirmed by the featureless surface roughness distributions across the fiber cleave shown in Fig. S3(e). While these results are encouraging, further studies are needed to quantify the presence and distribution of the air defects in the bulk of the fiber cores.


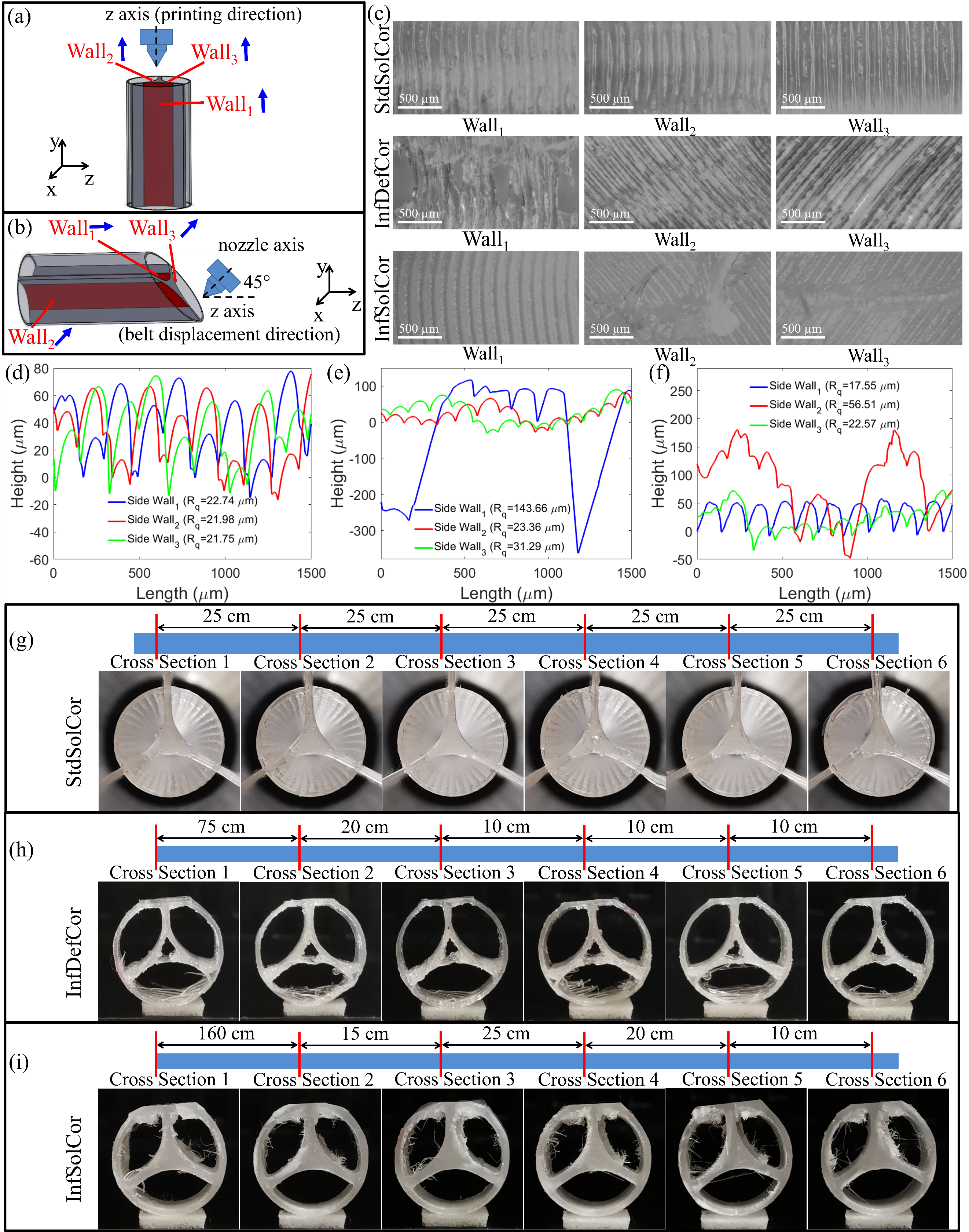


Figure S2. Schematic of the three concave walls (red regions) and their corresponding profiling directions (blue arrows) for (a) StdSolCor and (b) InfDefCor and InfSolCor fibers. (c) Microscopic images of the three concave walls for all three fibers. Representative surface roughness distributions along the three concave walls for (d) StdSolCor, (e) InfDefCor, and (f) InfSolCor fibers. Images of the fiber cross-sections and their corresponding locations along the fiber length for (g) StdSolCor, (h) InfDefCor, and (i) InfSolCor fibers.


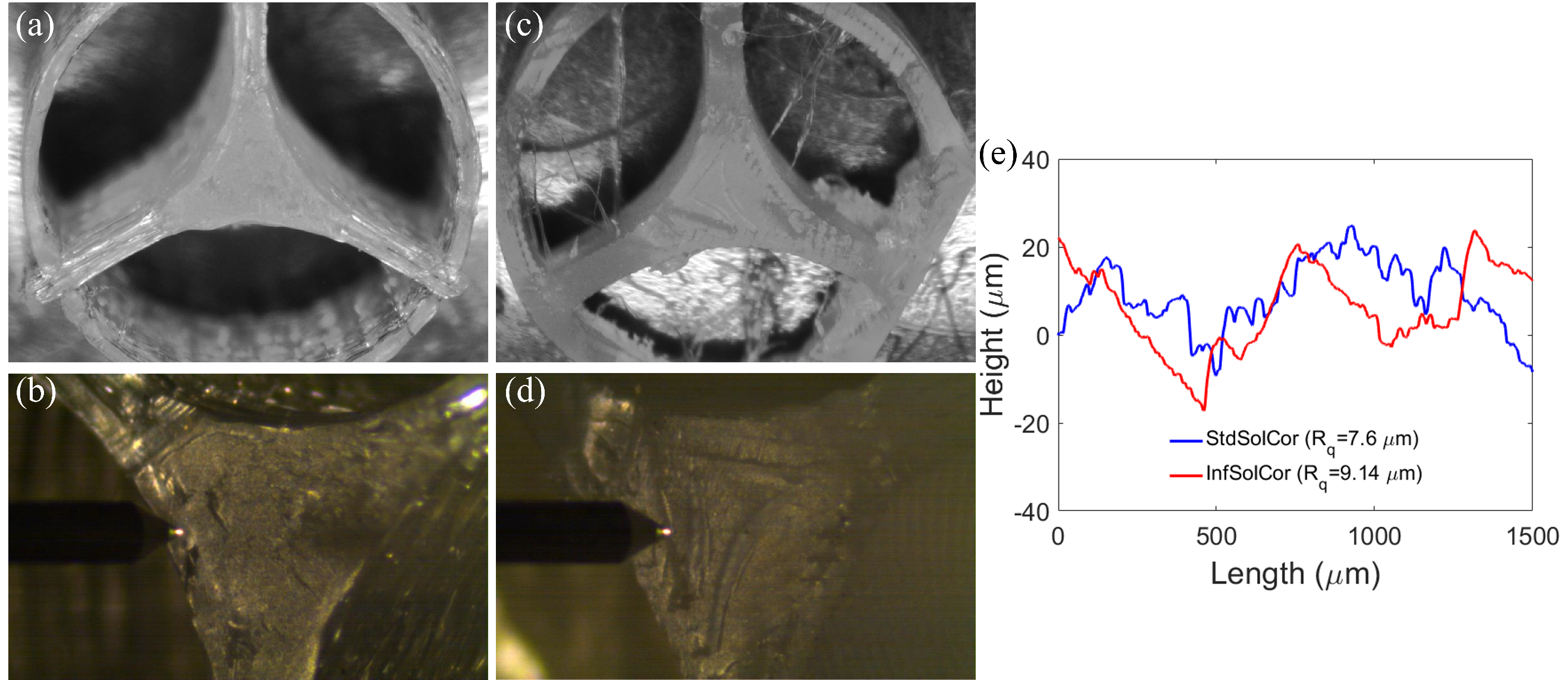


Figure S3. Microscopic images of the cleaved fibers after liquid nitrogen freezing and snapping, as well as microscopic images obtained from the Dektak profilometer of (a),(b) StdSolCor and (c),(d) InfSolCor fibers. (e) Surface roughness distribution across the cleaved surfaces of the two fiber cores.

# Section S4: Loss mechanisms in 3D printed fibers

When analyzing the effects of fabrication imperfections on fiber losses, one generally distinguishes the contributions of the core surface roughness from the bulk imperfections. Modeling the effects of surface roughness on fiber losses is not trivial as one has to resort to perturbation theory expressions or other advanced methods. At the same time, the effect of bulk imperfections is much easier to analyze. Indeed, one can use any convenient analytical form for the effective bulk material losses of core material, and then use a mode solver to find the corresponding losses of the fiber modes. By fitting the free parameters in the analytical loss model in such a way as to closely reproduce the experimentally measured fiber transmission losses. One can thus find the effective bulk losses of the fiber core material which, in our case, incorporate the effect of imperfections introduced by 3D printing.

While the loss fitting function can be of any complexity and it can contain many fitting parameters [an example of a 3-parameter function is given by Eq. (4)], for simplicity, in this work we want to use a function with only one fitting parameter as our focus is not on a rigorous understanding of the imperfections caused by the 3D printed process, but rather on a prospect of using 3D printing for THz fiber fabrication. One of the possible choices for the effective bulk material loss fitting function with one free parameter is just a constant. The physical meaning of such a constant is simply an average material loss over the spectral region used in the fitting. In our work, we choose the form of the fitting function to be proportional to the bulk PP material loss as measured in Ref. [14], with the constant of proportionality being the free parameter. In fact, this form of the fitting function only characterizes some average behavior of the core material losses over the spectral region of interest, and in this sense, it is no more or no less precise than just a constant considered in the previous example. At the same time, by definition, the form of the fitting function has an explicit reference to the ideal PP bulk material loss, thus allowing a more intuitive statement on the deterioration of the core material losses due to the effect of imperfections brought by the printing process.

On a general note, a comprehensive study on the nature of defects in the printed process is still needed to understand major loss mechanisms behind such fabrication techniques. However, the focus of this paper is not on the rigorous analysis of the 3D printing process itself, but rather on uncovering the potential of the infinity printing technique for THz fiber fabrication.

# Section S5: Excitation of a fiber featuring doubly degenerate modes

In the case of a fiber supporting a doubly degenerate fundamental mode, or an almost degenerate pair of modes, the electromagnetic fields at distance $z$ along the fiber length can be expressed as:

$$\begin{aligned} F\left( x,y,z \right)=C_{1}\cdot F_{1\left( x,y \right)}\cdot\exp\left( i\beta_{1}z \right)+C_{2}\cdot F_{2\left( x,y \right)}\cdot\exp\left( i\beta_{2}z \right)\#\left( S2 \right) \end{aligned}$$

where $F_{1\left( x,y \right)}$ and $F_{2\left( x,y \right)}$ are the transverse electromagnetic fields of the two modes, $C_{1}$ and $C_{2}$ are the excitation coefficients of the two modes at the fiber input ($z=0$), $\beta_{1}$ and $\beta_{2}$ are the propagation constant of the two modes. So, if the two modes are degenerate ($\beta_{1}=\beta_{2}=\beta$), the electromagnetic field propagating along the fiber will be:

$$\begin{aligned} F\left( x,y,z \right)={[C}_{1}\cdot F_{1\left( x,y \right)}+C_{2}\cdot F_{2\left( x,y \right)}]\cdot\exp\left( i\beta z \right)\#\left( S3 \right) \end{aligned}$$

Thus, a linear combination of the degenerate modes will be excited at the fiber input depending on the external excitation source. The total power carried by the modes will be the output power of the excitation source multiplied by $\left| C_{1} \right|^{2}+\left| C_{2} \right|^{2}$. The same argument holds if the fiber supports almost degenerate modes ($\beta_{2}-\beta_{1}=\Delta\beta\ll|\beta_{1}|$) which is the case of StdSolCor fiber. Then, the electromagnetic field propagating along the fiber will be:

$$\begin{aligned} F\left( x,y,z \right)={[C}_{1}\cdot F_{1\left( x,y \right)}+C_{2}\cdot F_{2\left( x,y \right)}\cdot exp(i\Delta\beta z)]\cdot\exp\left( i\beta_{1}z \right)\#\left( S4 \right) \end{aligned}$$

In this case, the two modes can be considered as degenerate if the propagation distance $z$is smaller than $L_{\Delta\beta}=\pi/{\Delta\beta}$, with the total excited power ~$\left| C_{1} \right|^{2}+\left| C_{2} \right|^{2}$ and transverse field distribution ~${[C}_{1}\cdot F_{1\left( x,y \right)}+C_{2}\cdot F_{2\left( x,y \right)}]$. In the case of experimental fibers InfDefCor and InfSolCor, the $L_{\Delta\beta}$ is calculated to be ~0.07 m and ~0.78 m which are shorter than the fiber lengths of 1.4 m and 2.5 m used in the measurements. This means that for practical applications it is preferable to excite only one of the two modes to mitigate the negative effects of the inter-modal beating, inter-modal dispersion, etc. that can affect fiber information transmission characteristics. To excite our fibers, we use a fixed WR-6 waveguide flange that supports a single fundamental mode. Therefore, before conducting any communication measurements, one has to find the optimal fiber orientations that result in the most efficient excitation of a single preferred fiber mode.

In the case of a StdSolCor featuring a symmetry reflection plane (ZOX), its modes can be characterized as being Y or X polarized. In this case, the excitation efficiency of a given mode is first optimized by aligning its principal polarization direction with that of a WR-6 mode. Then, by scanning the waveguide profile with a WR-6 flange along the symmetry axis, one finds the relative position of the two waveguides that maximize coupling efficiency. The same approach largely holds when the waveguide profile is almost symmetric as is the case of InfSolCor fiber shown in Fig. 3(a). Note that for a long-enough fiber, inter-modal scattering due to imperfections along the fiber length can lead to a significant power transfer between the modes of two distinct polarizations. This effect is especially pronounced in fibers that feature a doubly degenerate or a nearly degenerate fundamental mode as is the case for StdSolCor and InfSolCor [see Fig. 3(b)]. Once a particular polarization is excited, the fiber will maintain its polarization state only over a certain maximal propagation distance that depends strongly on the imperfection strength and is difficult to predict analytically.

In the general case of a non-symmetric profile [InfDefCor fiber or InfSolCor fiber shown in Fig. 3(a)], the two lowest order fiber modes can no longer be described as X or Y polarized. Thus, both the fiber orientation with respect to the polarization direction of the WR-6 modal field, as well as relative position of the two waveguides have to be optimized simultaneously to maximize coupling efficiency into a single fiber mode. Additionally, X-like modes show stronger than Y-like modes confinement in the lossy core, and as a consequence, they feature somewhat higher absorption losses, but at the same time somewhat lower bending and scattering losses than the Y-like modes. Moreover, for InfDefCor, anti-crossing occurs in the vicinity of a carrier frequency of 128 GHz for Y-like modes, which leads to high modal group velocity dispersion, and makes this mode undesirable. Anti-crossing between the Y-like core mode and one of the cladding modes manifests itself as “kinks” in the Y-like mode dispersion relation curve [see Fig. 3(b)], as well as in the Y-like mode loss curve [see Fig. 3(c)]. To be more rigorous, one has to plot dispersion relations of both modes to see a classic avoided crossing behavior near a phase-matching point between the dispersion relations of the two modes. Therefore, in our experimental studies, we favor X-like modes to the Y-like modes, especially for the InfDefCor.

# Section S6: Modal structure of the bent fiber

Here we present the theoretical studies of bending losses for the X-polarized and X-like modes of the three fibers [see Fig. 4(d)] for the two orthogonal fiber bending directions (parallel and perpendicular to the modal dominant polarization direction). As an example, the schematics of the two bending directions for the StdSolCor are shown in Fig. S4(a). There, the bend plane is either coincident with (left) or perpendicular to the X-axis (right), which is also the polarization direction of the StdSolCor X-polarized mode. The bending radius ($R_{b}$) is defined as the distance from the fiber center to the bend axis. The simulations for the complex dispersion relations of the modes of a bend are carried out using COMSOL Multiphysics (axis-symmetric 2D model) using the same fiber cross-section as in Fig. 1 while considering the fiber material as lossless.

In Figs. S4 (b,c) we show complex dispersion relation of the X-polarized and X-like modes at 128 GHz propagating through two different bend types with bending radii $R_{b}$ ranging from 10 mm to 30 mm. These values are chosen to study the degradation of the fiber performance under tight bending conditions that can be encountered in practical applications. We observe that the real part of the modal effective refractive indices as well as modal losses can increase considerably for tighter bends, more so in the case of the InfDefCor that features weaker modal confinement in the core. This is due to increased modal presence in the solid core, bridge and cladding regions for tighter bends as clearly seen from the modal field distributions presented in Figs. S4(d-f).

Moreover, we also find that bends that are perpendicular to the modal polarization direction have a smaller effect on the modal refractive index and losses than bends that are parallel to the modal polarization direction. This is related to anisotropy in the
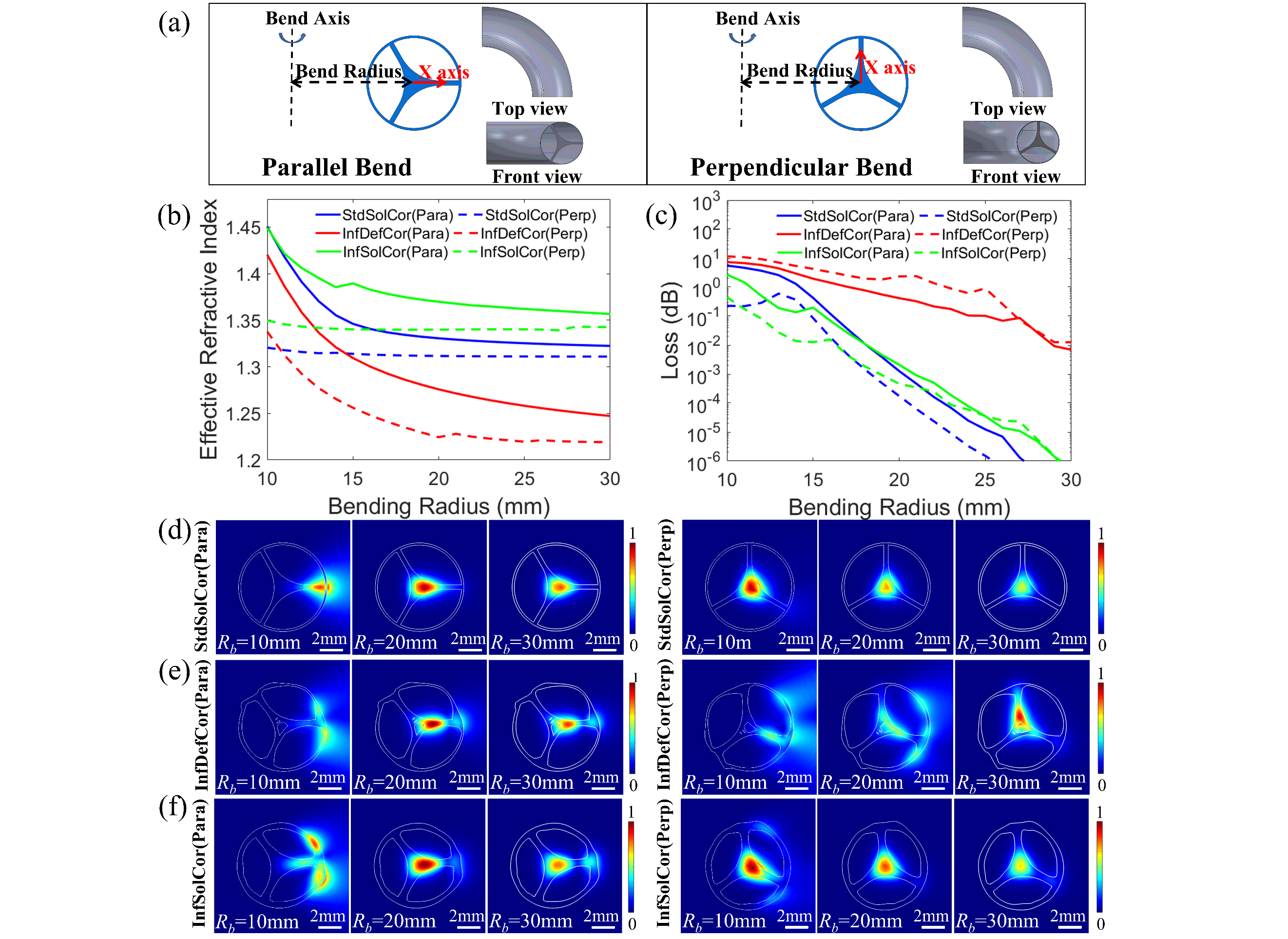


Figure S4. (a) Schematics of the two types of 90° bends with a 15 mm bending radius for StdSolCor. (b) Theoretical effective refractive indices and (c) bending losses per 90° bend (by power) of the X-polarized and X-like modes for the three fibers and the two types of bends as a function of the bending radius at 128 GHz. (d-f) Normalized electric field distributions of the X-polarized and X-like modes for the three fibers and the two types of bends as a function of the bending radius at 128 GHz.

fiber geometry. Particularly, for the perpendicular bends, the core boundary is almost perpendicular to the bend plane, and, as a consequence, modal fields are strongly confined in the fiber core by the high core/air refractive index contrast, thus preventing them from being pulled along the radial direction of the bend. In contrast, for parallel bends, the core boundary isparallel to the bend plane and nothing prevents the modal fields to be pulled along the bend radial direction into the bridge and cladding regions.

Finally, from Fig. S4 (c) we observe that for the StdSolCor and InfSolCor, bending losses smaller than ~0.1 dB per 90° bend for both bend types are achieved for bending radius larger than ~16 mm, while for InfDefCor one requires bending radius larger than ~27 mm. From this we conclude, that as far as bending losses are concerned, all the three fibers can readily tolerate tight bends with a radius as small as 3 cm, resulting only in a small loss increase. That said, we also note zero dispersion frequency of a bend mode will be somewhat shifted from that of a straight fiber. Also, additional losses are expected at the junction between a straight fiber and a bent section due to a mismatch in the modal field distributions of the two waveguides. Therefore, a complete analysis of the degradation of the information capacity of a fiber link with bends is complex and deserves a separate study, while results of this section should be rather considered as an indication of the robustness of our fibers to bending perturbations.

Note that in our estimations of the fiber bending losses (see Fig. S4) we assume that the bent fiber preserves the same cross-section regardless of the bending radius. In fact, in the case of tight bends, significant deformations or even breakage in the fiber structure can occur. Experimentally we find that InfSolCor fiber, for example, can be bent down to ~ 4 cm radius without visible deformations in its outer jacket, while breakage occurs at bending radii of ~3.5 cm (see Fig. S5 as well as Supplementary Video of the InfSolCor Fiber Bending Experiment). Fiber bending radii can be extracted from the images by inscribing as large a circle as possible that touches the fiber centerline (fitted by a parabola) at the apex. While these bending radii are adequate for most practical applications, if tighter bends are required, bend adapters can be printed in the shape of a fiber bend of constant cross-


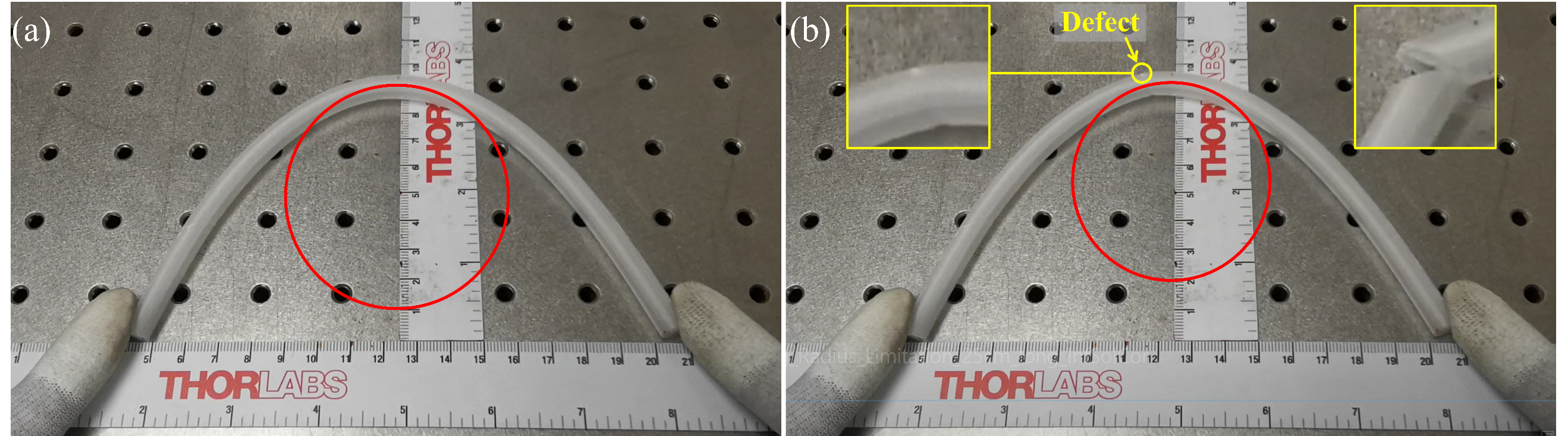


Figure S5. (a) Curved InfSolCor fiber of ~8 mm outer diameter with a bending radius at the apex of ~4 cm, no visible deformation of the fiber jacket is observed. (b) When further reducing fiber bend radius to ~3.5 cm, the defect appears in the outer cladding near the apex point, and the fiber snaps. Insets: enlarged views of the defect point (left) and the crack extending along the layer deposition direction (right).

section (as modeled in the paper) and then connectorized to the straight fibers. Therefore, theoretical predictions from Fig. S4 of the fiber bending loss at tighter bending radii (<~3 cm) should be rather considered in the context of separately printed bend adaptors. At the same time, further modeling is required (far beyond the scope of the paper) to understand bend-induced structural deformations and their effect on the optical properties in the infinity printed fibers.

# Section S7: Continuous wave THz spectroscopy system and mode field imaging


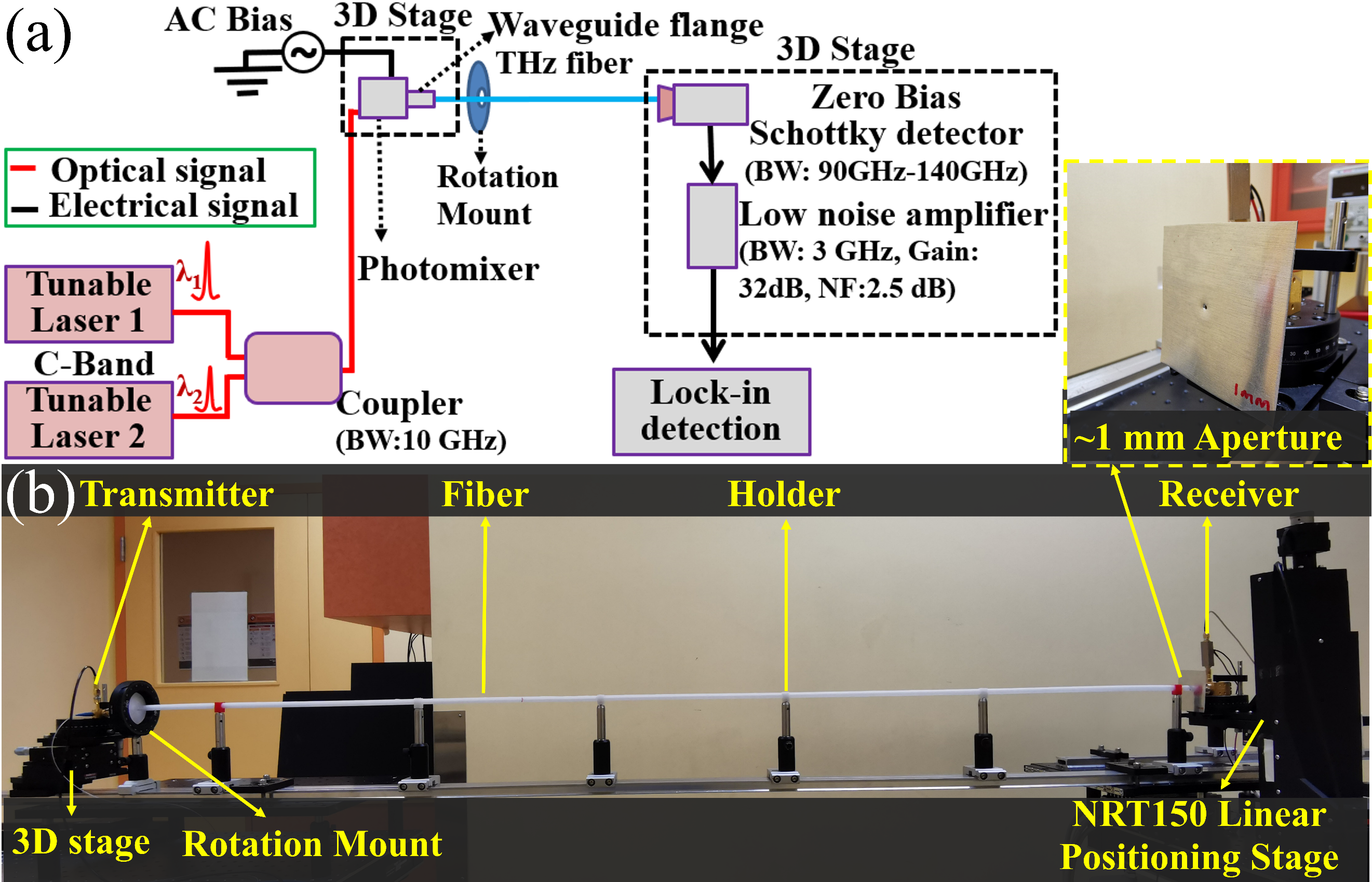


Figure S6. (a) Schematic of the CW THz spectroscopy system. (b) Experiment setup for the near-field THz modal imaging of InfSolCor (1.6 m). Inset: Enlarged view of the subwavelength (~1 mm) aperture mounted on the horn antenna.

The mode field imaging for all three fibers was carried out by using the Continuous Wave (CW) THz spectroscopy system. The system schematic and experiment setup are shown in Fig. S6. The CW THz spectroscopy system can be briefly described as follows: Two tunable lasers (TeraBeam) operating at judiciously mismatched wavelengths are used to optically drive the THz photomixer (emitter) for the difference frequency generation of THz waves. THz radiation from the photomixer (Model: IOD-PMD-14001from NTT Electronics Inc) is guided inside a WR-6 rectangular waveguide flange [Fig. 4(c)], which is butt-coupled to the fiber under study. On the receiving end, a 10.8 mm diameter horn antenna is used to collect THz waves, which are then detected and demodulated using a zero bias Schottky detector (Model: WR8.0 ZBD-F from Virginia diodes Inc). A high gain low noise amplifier (Model: SLNA-030-32-30-SMA from Fairview Microwave, Inc) is then used to amplify the received signal for further signal processing.

Before conducting the mode field imaging, the 3D printed fibers, the transmitter, and receiver antenna was in a similar arrangement to the modal loss measurement shown in Fig. S7 to maintain the optimal excitation arrangement [see Supplementary Materials Section S7 for details of the optimal excitation arrangement], the difference is that the receiver was mounted on a 3-dimensional computer-controlled linear stage featuring on-axis accuracy of 2 µm (NRT150 from Thorlabs, Inc) and a sub-wavelength aperture of ~1 mm diameter (see the Inset of Fig. S6) was attached to the 10.8 mm diameter horn antenna of the detector to work as a near-field probe. The fiber excitation arrangement (position and rotation) was maintained the same as the transmission loss measurements. The modal field profile was acquired by raster scanning of the fiber end with a spatial resolution of 0.3 mm, resulting in 41$\times$41 images, each covering a 12 mm$\times$ 12 mm area of the fiber cross-section.

# Section S8: Experiment setup for transmission loss measurement


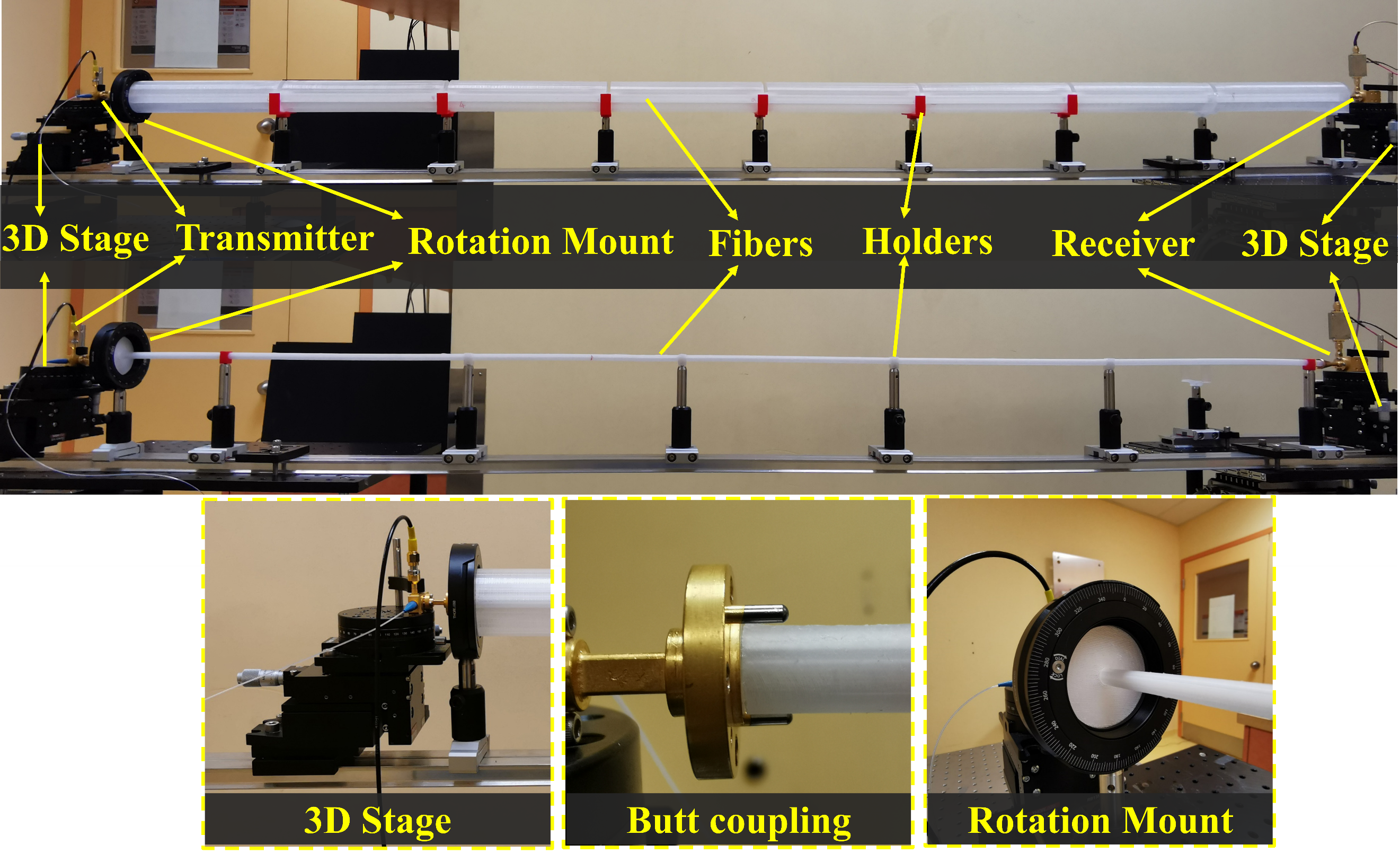


Figure S7. Experiment setup to measure the transmission losses for the StdSolCor (2 m) and InfSolCor (1.6 m). Inset: Enlarged view of the 3D stage, butt coupling of the InfSolCor with WR-6 rectangular flange, and the rotation mount.

The transmission loss measurements for all three fibers were characterized by using the CW THz spectroscopy system as shown in Fig. S6(a). For the experiment setup, at the transmitter end, the THz photomixer is butt coupled to the WR-6 rectangular flange. At the receiver end, the THz signals are captured by the horn antenna. The polarization directions of the transmitter and receiver antenna were fixed to be horizontal as we planned for the preferential excitation of the X-polarized and X-like modes of the 3D printed fibers in the experiments. Both the transmitter and receiver were mounted on the 3D Stage (RBL13M from Thorlabs, Inc) to finely optimize excitation and detection positions for the maximum detection signal. The fibers were mounted on a Rotation Mount (RSP2D from Thorlabs, Inc) at the input side to rotate the whole fiber with a minimal step value of 2 degrees for optimizing the excitation inclination. For each fiber inclination angle, both the transmitter and receiver were rescanned to maximize the signal. Finally, the measurement was conducted after the optimal fiber inclination and the positions of the transmitter and receiver were identified. To minimize the connector loss of StdSolCor, the fiber sections were mechanically joined using 3D-printed connectors shown in Fig. 2(e). Similarly, to minimize the effect of macro-bending in long fiber sections (InfDefCor and InfSolCor), multiple supports were fixed along the fiber length. After each measurement, the position of the receiver was reoptimized for the maximal output power. The position of the transmitter and the inclinations of all three fibers were fixed and unaltered throughout the loss measurements to maintain the optimal excitation arrangement (position and inclination).

We also note that in any CW THz spectroscopy setup with a layout similar to the one shown in Fig. S7, various types of standing waves will be excited that would reveal themselves as ripples in the measured spectra as can be clearly seen in Fig. 7. In fact, there are two principal types of standing waves in such systems. One is due to resonances in the cavity of optical length *L_s-d_* formed between the source and the detector, which results in spectral ripples of period *dν_s-d_~c/(2L_s-d_)*. In our experiments, the cavity size is ~0.25-2 m, thus resulting in ripples of different spectral sizes (~0.6-0.1 GHz) for fibers of different lengths. Such ripples are especially visible in the measurements with short fibers (0.25-0.75 m) shown in Fig. 7(a). In the visible and infrared spectral ranges, such standing waves can be suppressed by using optical isolators, which, unfortunately, is not the case in THz for the lack of optical isolators. Another type of standing wave is due to resonances in the cavity formed by a transmitter and an input facet of a fiber. Particularly, a photomixer cavity is coupled to fiber via a WR-6 rectangular waveguide of the total length of *L_t-f_* =~4-5 cm. Resonances in such a cavity are independent of the measured fiber length and result in spectral ripples with a period of *dν_t-f_~c/(2L_t-f_)* ~ 3 GHz that are clearly visible in our measurements [see, for example, Fig. 7(e)].

# Section S9: Photonics-based THz communication system and bit error measurements


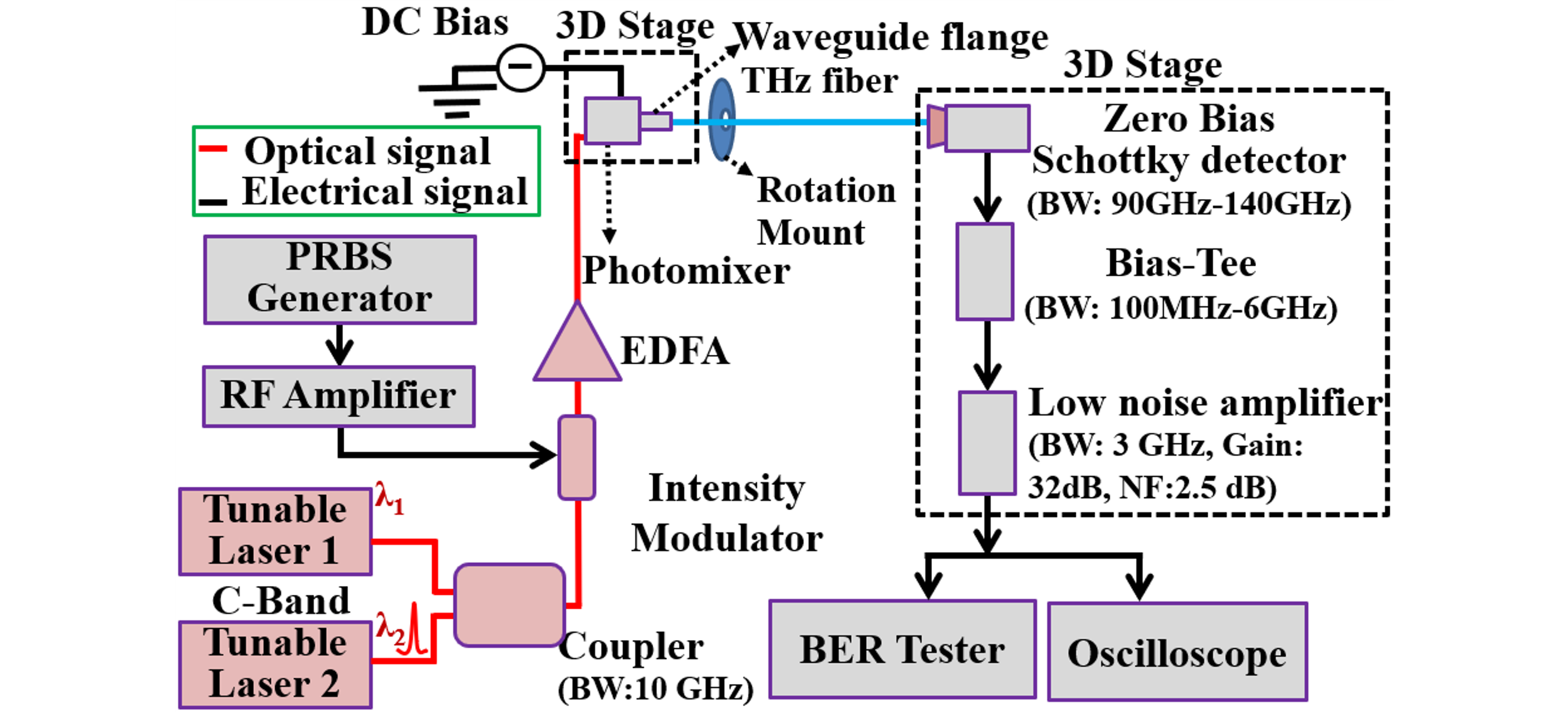


Figure S8. Schematic of the photonics-based THz communication system.

The bit error rate (BER) measurement was conducted with the photonics-based THz communication system. The schematic of the THz communication system is shown in Fig. S8, the infrared optical signals are modulated using an external electro-optic modulator (Model: LN81S-FC and MX10A from Thorlabs, Inc) in the transmitter section whereas the received and demodulated baseband signals are recorded/analyzed using a high-speed oscilloscope and BER tester (Model: MP2100B from Anritsu Corporation). The 3D printed fibers, the transmitter, and the receiver antenna were in a similar arrangement to the modal loss measurement (see Fig. S7), meanwhile, the communication unit was enabled and a Bias-Tee was added before the low noise amplifier at the receiver side for conducting the BER measurement. A non-return-to-zero (NRZ) pseudo-random bit sequence (PRBS) digital signal with a pattern length of 2^31^-1 was used as the baseband signal. The power of the THz transmitter antenna was set to -6.6 dBm (~218 µW), and the carrier frequency was chosen to be 128 GHz. The BER measurement was carried out by varying the bit rates from 1 Gbps to 6 Gbps. The target BER was set to 10^-12^ and the measurement duration is inversely proportional to the bit rate ($1/{(target BER\cdot bit rate})$). At each bit rate, the decision threshold was optimized to have a similar insertion (digital zero is mistaken as one) and omission errors (a digital one is mistaken for zero) ^21^.

In what follows we comment on the experimental Bit Error Measurements presented in Fig. 8.

Firstly, we note that it is somewhat surprising to see the StdSolCor fiber having a very steep BER slope compared to other fibers [left panel of Fig. 8(a)]. We believe that presented BER measurements were taken near the Zero Dispersion Frequency (ZDF) of the StdSolCor fiber (128 GHz) as suggested by our theoretical simulations shown in Fig. 5. Furthermore, we originally believed that the ZDF operation was also confirmed by the measurements shown in Fig. 8(b). Indeed, Fig. 8(b) shows the StdSolCor fiber optimal performance at 128 GHz, and a rapid decrease in the signal quality when deviating from this frequency by over ±2 GHz. Rapid degradation of the BER for the StdSolCor fiber at higher bit rates was interpreted as due to an increase of the signal bandwidth (proportional to the bitrate), thus, deviating from the ZDF operation for higher bitrates. That said, it is still somewhat strange to see such a rapid onset of dispersion effects when deviating from the ZDF by only several GHz. As shown in the theoretical plot in Fig. 5(b), reduction in the maximal bit rate away from the ZDF is indeed expected, however, it should be less dramatic than observed experimentally in Fig. 8(a). One of the reasons for such a fast degradation of the StdSolCor fiber signal quality away from ZDF could be the contribution to the signal dispersion of the resonant cavity at the fiber coupling end that has a free spectral range of 3 GHz (ripple spectral period). It is probably not accidental that for all the fibers shown in Fig. 8, BER starts deteriorating rapidly for bitrates higher than 3Gbps. At this point, however, we have no way of countering this issue as a spectral ripple is related to the internal design of a photomixer. In the absence of THz isolators, and a lack of alternative THz communications systems in the Montréal region we have to defer this issue to our future work.

Secondly, we would like to comment on the consistency between the fiber loss data (see Fig. 7) and an eye diagram (see Fig. 8). To avoid confusion, we note that the eye patterns that are shown in Fig. 8(a) do not have the same scales along the vertical direction (7 mV/division for StdSolCor and 4 mV/division for InfSolCor). In fact, the digital 1 signal level [Fig. 8(a)] for the StdSolCor fiber is always higher than that for the InfSolCor fiber, which is consistent with the fiber loss measurements of Fig. 7. Thus, as seen from Fig. 8(a), for example, at 3 Gbps the eye opening for the 2 m-long StdSolCor fiber is ~14 mV, while for the 1.6 m-long InfSolCor fiber it is only ~8 mV, while from the eye diagrams it looks like the two eye openings are similar, which is just an artifact due to difference is the vertical scales of the two figures.

Finally, we note that for Gaussian statistics, BER is an exponentially decreasing function^22^ of the ratio ${|V_{s}^{1,0}-V_{t}|}/{\delta V_{s}^{1,0}}$, where $V_{s}^{1,0}$ are the average voltages associated with either 0 or 1 levels, $V_{t}$ is the decision threshold voltage (for optimal performance, it is normally chosen in the middle of an eye opening), while $\delta V_{s}^{1,0}$ are the mean square deviations of the 0 or 1 signals from their mean due to Gaussian noise:

$$\begin{aligned} BER\left( V_{t} \right)=\frac{1}{2}\left( erfc\left( \frac{V_{s}^{1}-V_{t}}{\delta V_{s}^{1}} \right)+erfc\left( \frac{{V_{t}-V}_{s}^{0}}{\delta V_{s}^{0}} \right) \right)\#\left( S5 \right) \end{aligned}$$

At 3Gbps, the relative noise for the digital 1 (${\delta V_{s}^{1}}/{{(V}_{s}^{1}-V_{s}^{0})}$) in the StdSolCor fiber is almost twice as small as that for the InfSolCor fiber while having a near-Gaussian distribution. At the same time, the relative noise for the digital 0 (${\delta V_{s}^{0}}/{(V_{s}^{1}-V_{s}^{0})}$) for the StdSolCor fiber is similar to that of the InfSolCor fiber (however, it does not respect Gaussian distribution). These are the principal reasons why the BER for the StdSolCor fiber is much smaller than that for the InfSolCor fiber.”

# References

1 Takahashi, H. et al. in 2012 7th European Microwave Integrated Circuit Conference. 313-316 (IEEE).

2 Hirata, A. et al. 120-GHz-band wireless link technologies for outdoor 10-Gbit/s data transmission. IEEE Transactions on Microwave Theory Techniques **60**, 881-895 (2012).

3 Hirata, A. et al. in 2010 IEEE International Conference on Wireless Information Technology and Systems. 1-4 (IEEE).

4 Wu, Q. et al. in 2017 42nd International Conference on Infrared, Millimeter, and Terahertz Waves (IRMMW-THz). 1-2 (IEEE).

5 Dan, I. et al. A superheterodyne 300 GHz wireless link for ultra-fast terahertz communication systems. International Journal of Microwave Wireless Technologies **12**, 578-587 (2020).

6 Antes, J. et al. in 2012 IEEE/MTT-S International Microwave Symposium Digest. 1-3 (IEEE).

7 Shams, H. et al. in Broadband Access Communication Technologies XI. 101280G (International Society for Optics and Photonics).

8 Nagatsuma, T., Ducournau, G. & Renaud, C. C. Advances in terahertz communications accelerated by photonics. Nature Photonics **10**, 371-379, doi:10.1038/nphoton.2016.65 (2016).

9 Seeds, A. J., Shams, H., Fice, M. J. & Renaud, C. C. TeraHertz Photonics for Wireless Communications. Journal of Lightwave Technology **33**, 579-587, doi:10.1109/jlt.2014.2355137 (2015).

10 Akkaş, M. A. Terahertz wireless data communication. Wireless Networks **25**, 145-155, doi:10.1007/s11276-017-1548-4 (2017).

11 Rappaport, T. S. et al. Wireless communications and applications above 100 GHz: Opportunities and challenges for 6G and beyond. IEEE access **7**, 78729-78757 (2019).

12 Ma, J., Shrestha, R., Moeller, L. & Mittleman, D. M. Invited article: Channel performance for indoor and outdoor terahertz wireless links. APL Photonics **3**, 051601 (2018).

13 Rappaport, T. S. et al. Millimeter wave mobile communications for 5G cellular: It will work! IEEE access **1**, 335-349 (2013).

14 Kleine-Ostmann, T. & Nagatsuma, T. A Review on Terahertz Communications Research. Journal of Infrared, Millimeter, and Terahertz Waves **32**, 143-171, doi:10.1007/s10762-010-9758-1 (2011).

15 Elayan, H., Amin, O., Shihada, B., Shubair, R. M. & Alouini, M.-S. Terahertz Band: The Last Piece of RF Spectrum Puzzle for Communication Systems. IEEE Open Journal of the Communications Society **1**, 1-32, doi:10.1109/ojcoms.2019.2953633 (2020).

16 Castro, C., Elschner, R., Merkle, T., Schubert, C. & Freund, R. Experimental Demonstrations of High-Capacity THz-Wireless Transmission Systems for Beyond 5G. IEEE Communications Magazine **58**, 41-47 (2020).

17 Castro, C., Elschner, R., Merkle, T., Schubert, C. & Freund, R. in 2020 Third International Workshop on Mobile Terahertz Systems (IWMTS). 1-4 (IEEE).

18 Shaw, J. A. Radiometry and the Friis transmission equation. American journal of physics **81**, 33-37 (2013).

19 Ung, B., Mazhorova, A., Dupuis, A., Roze, M. & Skorobogatiy, M. Polymer microstructured optical fibers for terahertz wave guiding. Opt Express **19**, B848-861, doi:10.1364/OE.19.00B848 (2011).

20 Carneiro, O. S., Silva, A. F. & Gomes, R. Fused deposition modeling with polypropylene. Materials & Design **83**, 768-776, doi:10.1016/j.matdes.2015.06.053 (2015).

21 Nallappan, K. et al. Dispersion Limited versus Power Limited Terahertz Transmission Links Using Solid Core Subwavelength Dielectric Fibers. Photonics Research **8**, 1757-1775, doi:10.1364/prj.396433 (2020).

22 Bergano, N. S. Undersea amplified lightwave systems design. (1997).

# Acknowledgments

We thank technician Mr. Jean-Paul Lévesque and Mr. R.W.H. Theunissen (BlackBelt 3D BV Inc.) for their assistance.

# Author contributions statement

G.X, K.N., and M.S. conceived the study; K.N and M.S. supervised the study. G.X. performed all the theoretical simulations and experimental characterizations and prepared all figures. G.X, K.N., and M.S. wrote the manuscript. All authors reviewed the manuscript.

# Competing interests

The authors declare that they have no competing interests.
